# Manganese Porphyrin‐Catalyzed One‐Pot Oxidation of Lupeol: Efficient Conversion of Its Terminal Alkene Into Carboxylic Acids

**DOI:** 10.1002/cbdv.71239

**Published:** 2026-05-04

**Authors:** Leila Renan Oliveira, Pedro Fonseca‐Pinheiro, Lucienir Pains Duarte, Diogo Montes Vidal, Grasiely Faria de Sousa, Dayse Carvalho da Silva Martins

**Affiliations:** ^1^ Departamento de Química Instituto de Ciências Exatas Universidade Federal de Minas Gerais Belo Horizonte Minas Gerais Brazil

**Keywords:** biomimetic systems, catalysis, natural products, P450 cytochromes, triterpene

## Abstract

The PhI(OAc)_2_‐oxidation of the triterpene lupeol by manganese porphyrin was achieved, leading to 94% of lupeol conversion. Conditions aligned with principles of green chemistry, including the use of ethyl acetate as a solvent and iodobenzene diacetate as an oxidant, were studied. This approach led to the production of eight products from lupeol, notably achieving transformations at the C20 olefin moiety; six lupeol's products were isolated whereas the other two had their structures proposed supported on basic chemical tests (e.g., Fehling's test) and well‑established reactivity patterns in organic chemistry. These modifications were performed without the typical preceding protection of the C3 hydroxyl of lupeol, simplifying the synthetic route. Besides, the isopropenyl group of lupeol was converted to carboxylic acids through one‐pot oxidation. This study reinforces the promising use of manganese porphyrins as catalysts for the oxidative transformation of other natural products of biological interest.

## Graphical abstract

Manganese porphyrin catalyst enables green PhI(OAc)_2_ oxidation of lupeol in ethyl acetate, achieving 94% conversion and the formation of eight products, including selective C20 olefin transformations and one‐pot conversion of the isopropenyl group to carboxylic acids, all without the need of C3‐OH protection step. The manganese porphyrin act as versatile catalysts for sustainable oxidative transformation of a complex natural product.

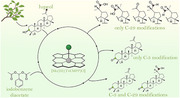

## 1 Introduction

The use of natural products in quotidian life has spread all over the world. A wide range of chemical compounds have been extracted from a variety of plants and their different parts (bark, flower, leaf, root, seed, seed oil, shoot, and stem), and they have presented a broad spectrum of biological activities, including antimicrobial, anticancer, antioxidant, anti‐inflammatory, anti‐diabetic, immunomodulatory, anti‐insecticidal, and antiallergic [1, 2]. Among all chemicals obtained from plants, terpenes and terpenoids have been extensively studied. Terpenes are described as compounds with simple hydrocarbon structures, while terpenoids are described as a modified class of terpenes with different functional groups and oxidized methyl groups moved or removed at various positions [3].

**SCHEME 1:**
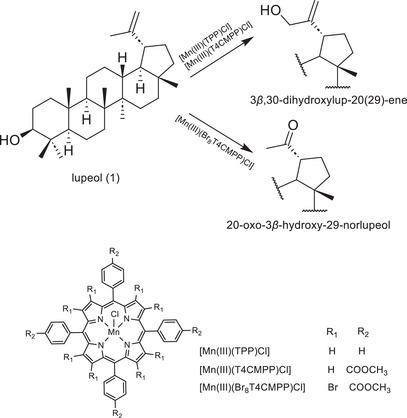
Oxidation of lupeol by iodosylbenzene (PhIO) in 1,2‐dichloroethane, catalyzed by different Mn‐porphyrins. Conditions: lupeol (0.33 mmol), Mn‐porphyrin (0.0099 mmol), and PhIO (0.40 mmol), in a molar ratio of MnP:lupeol:PhIO = 1:33:40, for 4 h at 0°C [19].

Lupeol (1, Scheme 1), a pentacyclic triterpenoid, has been extracted from many medicinal plants, such as in leaves of *Maytenus salicifolia* Reissek (Celastraceae). Sohag et al. summarized several natural sources of lupeol [4]. This compound has presented several biological activities, such as antioxidant, anti‐inflammatory, anticancer, anti‐diabetic, nephroprotective, neuroprotective, dermoprotective, osteoprotective, and cardioprotective [4, 5, 6, 7, 8]. In addition to the various studies that show the potential use of lupeol, numerous studies have highlighted the activity of its derivatives, with emphasis on the esters obtained in position 3 of this triterpene [8, 9]. Lupeol has also been explored as a starting material in semisynthetic approaches to provide novel compounds with structural modifications in the double bond at C20 and C29 [10, 11, 12], which usually needs to be preceded by protection of the C3 hydroxyl. The need to protect the C3 hydroxyl group usually introduces considerable complications, additional steps, and waste to the synthesis processes, making it difficult to efficiently obtain these biologically active compounds. Lupeol derivatives via modification at C29 or C20 positions have shown some important biological activities such as antitrypanosomal [13], antileukemic [14], antitumor [15], antioxidant [16], reduces NO production in BV‐2 cells [17], and antileishmanial [18]. Frequently, these modifications require prior protection of the C3 hydroxyl group, increasing the complexity and the steps to the experimental process.

In a previous study [19], the oxidation of lupeol using iodosylbenzene (PhIO) as an oxidant mediated by three manganese porphyrins as P450 biomimetic models was described. The reactions were carried out under mild conditions and selectively yielded 3*β*,30‐dihydroxylup‐20(29)‐ene or 20‐oxo‐3*β*‐hydroxy‐29‐norlupeol (Scheme 1), which have difficult access via conventional organic routes. While the selective production of compounds with modifications restricted to the C20 position of lupeol has been demonstrated, further efforts are currently underway to enhance the system's efficiency, with a focus on incorporating principles of green chemistry to improve its environmental sustainability [20].

Herein we describe the PhI(OAc)_2_‐oxidation of lupeol by a manganese porphyrin, the one which led to the greatest degree of transformation of lupeol in the previous work [19], [Mn(III)(T4CMPP)Cl], and it was possible to transform 94% of lupeol producing eight different products including three acyl derivatives at the C20, two of them keeping the hydroxyl at C3 intact. Six of these products were isolated and characterized, whereas the other two had their structures proposed based on chemical tests (e.g., Fehling's test) and well‑established reactivity patterns in organic chemistry. This one‐pot functionalization at C20 position (without protecting C3) is an advantage of our biomimetic approach, representing a way to simplify and optimize the synthesis process. To the best of our knowledge, there has been no report on the biomimetic oxidation of lupeol other than ours [19], which drives us to continue studies on the oxidation of this triterpene by porphyrinic catalysts.

## 2 Experimental Section

### 2.1 General Information

The catalyst 5,10,15,20‐tetrakis(4‐carbomethoxyphenyl)porphyrin‐21,23‐diidomanganese(III) chloride, [Mn(III)(T4CMPP)Cl], was prepared and purified as previously reported [21, 22]. This compound exhibited characterization features that are consistent with the published data. The commercial iodobenzene diacetate [PhI(OAc)_2_, 98%; Aldrich], ethyl acetate (HPLC grade, Vetec), sodium sulfite (Na_2_SO_3_; >98%; Neon), sodium borate or borax (Na_2_B_4_O_7_·10H_2_O, 99%, Aldrich), and sodium bicarbonate (NaHCO_3_; >99,7%; Vetec) were used without treatment. Lupeol was isolated from species of the Celastraceae family by several chromatographic columns using silica gel and hexane/chloroform (7:3). Extracted lupeol was purified by recrystallization in ethanol with a few drops of chloroform, which yielded a highly pure compound, whose characterization features are consistent with the published data [23].

Thin‐layer chromatography (TLC) was carried out on plates coated with silica gel 60 using a solution of 1:1 v/v vanillin (vanillin, absolute ethanol, 1% m/v) and perchloric acid (3% v/v), followed by heating to 100°C. Column chromatography (CC) was carried out using Sephadex LH‐20 or silica gel 60 (230–400 mesh) as the stationary phase. Hexane, chloroform, ethyl acetate, and methanol were used as eluents, either pure or in increasing polarity mixtures. Preparative layer chromatography (PLC) was carried out in aluminum plates coated with silica gel 60 (10 × 20 cm), containing a 254 nm fluorescence indicator, and a thickness of 1 mm. The eluents used were hexane, dichloromethane, chloroform, and ethyl acetate, either pure or in mixture, depending on the sample. Detection methods were fluorescence under UV light at 254 nm or 360 nm, as well as by spraying a revealing solution (1:1 vanillin and perchloric acid) on the trimmed edge of the plate.

1D NMR data were obtained on a Bruker Avance DRX‐600 spectrometer using CDCl_3_ as solvent and tetramethylsilane (TMS; *δ*_H_ = *δ*_C_ = 0) as internal standard. Chemical shifts were measured in parts per million (ppm) and coupling constants (*J*) were calculated in Hz. Ultraviolet‐visible (UV–vis) spectra (190–1100 nm) were recorded on the HP‐8453A diode‐array spectrophotometer. Infrared spectra were obtained using a Shimadzu IR‐408 spectrometer (Shimadzu, Kyoto, Japan) with KBr pellet samples or a Perkin Elmer, Spectrum One spectrophotometer (ATR). Infrared spectrum (ATR) of P1 was obtained using a Shimadzu IRSpirit (Shimadzu, Kyoto, Japan) spectrophotometer.

The GC‐MS analyses were carried out using a Shimadzu GC‐2010 chromatograph, with an RTX‐5MS (30m x 0.25 mm with 20 µm film thickness) coupled with a Shimadzu QP2010 SE spectrometer (70 eV). Two distinct methods were used. In the first one, the initial column temperature was 100°C, using a 10°C min^−1^ heating ramp until 325°C, holding the temperature for 20 min. The injection and interface temperatures were 325°C, whilst the ion source temperature was 250°C. 1 µL aliquots of the dilute reactions were injected in *split* mode (1:10), using He as the mobile phase. In the second one, the initial column temperature was 100°C, using a 7°C min^−1^ heating ramp until 310°C, holding the temperature for 30 min. The injection and interface temperatures were 250°C, whilst the ion source temperature was 200°C. 1 µL aliquots of the dilute reactions were injected in *split* mode (1:20), using He as the mobile phase. The NIST Mass Spectrometry Data Center and Flavors and Fragrances of Natural and Synthetic Compounds MS libraries were used for fragment identification. The high‐resolution mass spectra were acquired in a ThermoScientific Q‐Exactive mass spectrometer (ThermoScientific, Waltham, USA) using H‐ESI as an ionization source.

To confirm the presence of aldehyde, a Fehling's solution was used. Fehling's solution was prepared using an aqueous solution of copper(II) sulfate pentahydrate (Fehling's A) and an aqueous solution of potassium sodium tartrate and sodium hydroxide (Fehling's B). A few drops of the reaction or control mixture were added to Fehling's solution in a test tube. The tube was then heated using a heat blower, and if a reddish‐brown solid precipitated, the result was considered positive; otherwise, it was considered negative.

### 2.2 Oxidation Reactions

The reactions were performed based on Martins et al. [19] with modifications. [Mn(III)(T4CMPP)Cl] (0.0014 mmol) and PhI(OAc)_2_ (0.14 mmol) were weighed in a 10 mL glass vial, and 1 mL of ethyl acetate was added alongside 1 mL of a 20 mg mL^−1^ solution of lupeol (0.047 mmol) in ethyl acetate. The reaction was kept under magnetic stirring at 50°C for 24 h, being quenched with the addition of 50 µL of a saturated aqueous solution of Na_2_SO_3_/Borax and 50 µL of ethyl acetate. Sodium sulfite acts as a rapid reducing agent, reducing the PhI(OAc)_2_ to iodobenzene (PhI), while borax functions as a buffering base to maintain a controlled, mildly basic medium, accounting for acetate formation in the medium. 50 µL of the quenched reaction was diluted to 1 µL with ethyl acetate and analyzed with GC‐MS, through comparison with a stock solution of lupeol. Control reactions, in the absence of either [Mn(III)(T4CMPP)Cl] or PhI(OAc)_2_, were performed and analyzed in the same manner. For all cases, the conversion was calculated from the integrated lupeol peak area at time t relative to the corresponding t_0_ reference aliquot, injected at the same dilution and under identical GC conditions. Lupeol conversion (%) = 100% × [1 − (Area_Lupeol(end)_/Area_Lupeol(beginging)_)]. To minimize injection‐to‐injection variability, we used identical solvent and dilution, constant split ratio and injection volume, fixed integration parameters, and replicate injections. The destruction degree of the catalyst was determined by UV–vis absorption spectroscopy by monitoring the molar absorptivity (ε) of the *Soret* band of the catalyst.

After the first GC‐MS analysis, 23 separate reactions were performed following the same procedure, exactly as described above. After 24 h, the content of all reactions was put together, and the solvent was removed using a rotary evaporator.

The resulting material was submitted to column chromatography (CC) using Sephadex LH‐20 and a mixture of hexane/chloroform/methanol (2:1:1) as eluent to remove the catalyst and oxidant byproduct. The resulting fractions were grouped according to the TLC profile, and the triterpenes containing group (354 mg) was submitted to CC using silica gel 60 (230‐400 mesh). When necessary, the resulting fractions were purified by preparative layer chromatography (PLC). Product amounts were determined from the isolated mass after purification. Isolated yield (IY) for each product was calculated using the equation IY(%) = 100% × (m_x_/M_x_)/n_0_, in which m_x_ is the isolated mass (g) for each product, M_x_ is the molecular weight (g mol^−1^) of each compound, and n_0_ is the initial amount (mol) of lupeol at the beginning of the reaction.

*Lupenone* **(P1; 9.5 mg; yield: 2%)**: **^1^H NMR (400 MHz, CDCl_3_) *δ***: 2.49 (m, 1H, H‐2), 2.39 (m, 2H, H‐2, H‐19), 1.03 (s, 3H, H‐24), 0.93 (s, 3H, H‐25), 1.07 (s, 6H, H‐23, H‐26), 0.96 (s, 3H, H‐27), 0.80 (s, 3H, H‐28) 4.69 (s, 1H, H‐29), 4.57 (s, 1H, H‐29), 1.69 (s, 3H, H‐30). **^13^C NMR (100 MHz, CDCl_3_) *δ***: 39.6 (C1), 34.2 (C2), 218.3 (C3), 47.4 (C4), 54.9 (C5), 19.7 (C6), 33.6 (C7), 40.8 (C8), 49.8 (C9), 36.9 (C10), 21.5 (C11), 25.2 (C12), 38.2 (C13), 42.9 (C14), 27.4 (C15), 35.5 (C16), 43.0 (C17), 48.3 (C18), 48.0 (C19), 150.9 (C20), 29.8 (C21), 40.0 (C22), 26.7 (C23), 21.0 (C24), 16.0 (C25), 15.8 (C26), 14.5 (C27), 18.0 (C28), 109.4 (C29), 19.3 (C30). **IR (ATR) *ν*/cm^−1^**: 2,940; 2,856; 1,704; 1,644; 1,456; 1,381; 870; 581. **ESI‐MS *m/z* (rel. int.)**: 109 (100); 205 (96); 95 (93); 81 (82); 107 (72); 121 (69); 93 (65); 123 (58); 69 (57); 55 (57); 67 (55); 108 (51); 41 (49); 135 (47); 79 (45); 149 (42); 42 (42); 189 (41); 119 (38); 122 (35); 147 (34); 204 (34); 105 (34); 133 (33); 203 (32); 161 (30); 91 (30); 218 (29); 94 (29); 175 (25); 205 (24); 136 (24); 245 (24); 110 (24); 424 (24); 163 (24); 313 (24); 137 (23); 83 (23); 68 (21); 134 (21); 148 (20); 82 (20); 97 (17); 120 (17); 96 (17); 409 (17); 177 (16); 314 (16); 191 (15); 232 (15); 111 (15); 217 (15); 77 (13); 125 (13); 162 (13); 219 (12); 150 (12); 106 (12); 190 (12); 145 (11); 176 (11); 57 (10).

*29‐norlupan‐3,20‐dione* **(P2; 5.2 mg; yield: 1%)**: **^1^H NMR (600 MHz, CDCl_3_) *δ***: 2.48 (ddd, *J* = 16.6; 9.5; 7.5 Hz, 1H, H‐2), 2.41 (m, 1H, H‐2), 2.59 (td, *J* = 11.3; 11.3; 6.0 Hz, 1H, H‐19), 1.07 (s, 3H, H‐23), 1.03 (s, 3H, H‐24), 0.92 (s, 3H, H‐25), 1.06 (s, 3H, H‐26), 0.98 (s, 3H, H‐27), 0.79 (s, 3H, H‐28), 2.16 (s, 3H, H‐30). **^13^C NMR (150 MHz, CDCl_3_) *δ***: 39.8 (C1), 34.1 (C2), 218.2 (C3), 47.3 (C4), 54.8 (C5), 19.7 (C6), 33.5 (C7), 40.7 (C8), 49.5 (C9), 36.9 (C10), 21.5 (C11), 27.3 (C12), 37.1 (C13), 42.8 (C14), 27.2 (C15), 34.9 (C16), 43.0 (C17), 49.6 (C18), 52.6 (C19), 212.8 (C20), 27.7 (C21), 39.5 (C22), 26.7 (C23), 21.0 (C24), 16.0 (C25), 15.7 (C26), 14.4 (C27), 18.0 (C28), 29.2 (C30). **ESI‐MS *m/z* (rel. int.)**: 163 (100); 382 (84); 162 (76); 81 (68); 95 (54); 205 (51); 55 (49); 107 (48); 67 (46); 147 (43); 43 (42); 109 (41); 93 (40); 121 (40); 69 (40); 41 (38); 149 (34); 148 (34); 79 (33); 135 (30); 119 (28); 105 (27); 383 (26); 123 (26); 187 (25); 133 (25); 125 (23); 91 (22); 161 (22); 176 (21); 164 (18); 177 (18); 83 (18); 367 (17); 137 (17); 134 (16); 189 (16); 97 (15); 94 (14); 206 (14); 145 (14); 122 (14); 120 (13); 96 (13); 108 (13); 175 (13); 203 (12); 136 (11); 191 (11); 82 (11); 150 (11); 273 (10); 440 (1).

*3β‐hydroxy‐29‐norlupan‐20‐one* **(P3; 8.2 mg; yield: 2%)**: **^1^H NMR (400 MHz, CDCl_3_) *δ***: 3.19 (dd, *J* = 11.3; 4.9 Hz, 1H, H‐3), 2.58 (td, *J* = 11.3; 11.3; 5.9 Hz, 1H, H‐19), 0.83 (s, 3H, H‐25), 0.76 (s, 3H, H‐24), 1.02 (s, 3H, H‐26), 0.97 (s, 6H, H‐23, H‐27), 0.77 (s, 3H, H‐28), 2.15 (s, 3H, H‐30). **^13^C NMR (100 MHz, CDCl_3_) *δ***: 38.8 (C1), 27.5 (C2), 79.1 (C3), 39.0 (C4), 55.4 (C5), 18.4 (C6), 34.3 (C7), 40.9 (C8), 50.4 (C9), 37.3 (C10), 21.0 (C11), 27.3 (C12), 37.2 (C13), 42.8 (C14), 27.5 (C15), 35.1 (C16), 43.2 (C17), 49.8 (C18), 52.8 (C19), 213.1 (C20), 27.8 (C21), 40.0 (C22), 28.1 (C23), 15.5 (C24), 16.2 (C25), 16.1 (C26), 14.6 (C27), 18.1 (C28), 29.3 (C30). **IR (KBr) *ν*/cm^−1^**: 3,548; 3,472; 3,238; 2,940; 2,864; 1,638; 1,618; 1,456; 1,382; 622; 486. **ESI‐MS *m/z* (rel. int.)**: 189 (100); 81 (81); 95 (79); 384 (77); 43 (68); 135 (65); 207 (63); 107 (61); 176 (58); 121 (58); 55 (55); 69 (55); 93 (54); 67 (51); 162 (51); 109 (50); 119 (45); 147 (44); 41 (44); 187 (44); 123 (39); 149 (38); 163 (38); 79 (38); 148 (38); 136 (36); 190 (35); 133 (34); 105 (32); 161 (26); 175 (25); 91 (24); 385 (24); 177 (23); 94 (22); 122 (22); 83 (21); 134 (21); 203 (20); 71 (19); 57 (19); 108 (18); 120 (17); 96 (17); 208 (17); 191 (16); 137 (16); 82 (16); 97 (16); 164 (14); 145 (14); 188 (12); 111 (11); 275 (11); 68 (11); 369 (11); 106 (10); 173 (10); 442 (1).

*(20S)‐3‐oxolupan‐29‐oic acid* **(P4; 8.9 mg; yield: 2%)**: **^1^H NMR (400 MHz, CDCl_3_) *δ***: 2.51 (ddd, *J* = 15.6; 9.9; 7.5, 1H, H‐2), 2.41 (m, 2H, H‐2, H‐19), 2.79 (qd, *J* = 6.8; 6.8; 6.8; 3.4 Hz, 1H, H‐20), 1.05 (s, 3H, H‐23), 1.08 (s, 3H, H‐24), 0.95 (s, 3H, H‐25), 1.08 (s, 3H, H‐26), 0.95 (s, 3H, H‐27), 0.79 (s, 3H, H‐28) 1.06 (d, *J* = 7.0, 3H, H‐30). **^13^C NMR (100 MHz, CDCl_3_) *δ***: 39.7 (C1), 34.3 (C2), 218.3 (C3), 47.5 (C4), 55.1 (C5), 19.8 (C6), 33.8 (C7), 41.0 (C8), 49.5 (C9), 37.0 (C10), 21.5 (C11), 26.5 (C12), 38.0 (C13), 43.2 (C14), 27.4 (C15), 35.5 (C16), 43.2 (C17), 47.3 (C18), 40.3 (C19), 41.0 (C20), 23.8 (C21), 40.5 (C22), 21.2 (C23), 26.8 (C24), 16.0 (C25), 15.9 (C26), 14.4 (C27), 18.1 (C28), 180.8 (C29), 9.8 (C30). **IR (KBr) *ν*/cm^−1^**: 3,556; 3,474; 3,416; 3,238; 2,932; 2,860; 1,704; 1,638; 1,618; 1,458; 1,384; 622; 482.

*(20S)‐3β‐hydroxylupan‐29‐oic acid* **(P5; 5.8 mg; yield: 1%)**: **^1^H NMR (400 MHz, CDCl_3_) *δ***: 3.20 (dd, *J* = 11.2; 5.0 Hz, 1H, H‐3), 2.80 (qd, *J* = 6.9; 6.9, 6.9, 3.3 Hz, 1H, H‐20), 0.84 (s, 3H, H‐23), 0.93 (s, 3H, H‐24), 0.77 (s, 3H, H‐25), 1.06 (s, 3H, H‐26), 0.97 (s, 3H, H‐27), 0.78 (s, 3H, H‐28), 1.06 (d, *J* = 7.0 Hz, 3H, H‐30). **^13^C NMR (100 MHz, CDCl_3_) *δ***: 38.8 (C1), 27.4 (C2), 79.2 (C3), 37.3 (C4), 55.4 (C5), 18.5 (C6), 34.5 (C7), 39.0 (C8), 50.2 (C9), 37.9 (C10), 20.9 (C11), 26.5 (C12), 37.9 (C13), 43.2 (C14), 27.5 (C15), 35.6 (C16), 43.2 (C17), 47.4 (C18), 40.3 (C19), 41.0 (C20), 23.8 (C21), 40.5 (C22), 28.1 (C23), 15.5 (C24), 16.2 (C25), 16.1 (C26), 14.5 (C27), 18.1 (C28), 180.8 (C29), 9.8 (C30). **IR (KBr) *ν*/cm^−1^**: 3,552; 3,474; 3,414; 3,240; 2,940; 2,866; 1,638; 1,618; 622; 486. **HR‐H‐ESI‐MS** *m/z* 457.36872 [M]^−^ (calcd for C_30_H_49_O_3_ 457.37)

*(20R)‐3β‐hydroxylupan‐29‐oic acid* **(P6; 14.3 mg; yield: 3%)**: **^1^H NMR (600 MHz, CDCl_3_) *δ***: 3.21 (dd, *J* = 11.5; 4.5 H, 1H, H‐3), 2.80 (m, 1H, H‐20), 0.85 (s, 3H, H‐23), 0.92 (s, 3H, H‐24), 0.75 (s, 3H, H‐25), 1.04 (s, 3H, H‐26), 0.97 (s, 3H, H‐27), 0.77 (s, 3H, H‐28), 1.15 (d, *J* = 7.0 Hz, 3H, H‐30). **^13^C NMR (150 MHz, CDCl_3_) *δ***: 38.8 (C1), 27.4 (C2), 79.2 (C3), 41.0 (C4), 55.4 (C5), 18.5 (C6), 34.5 (C7), 39.0 (C8), 50.1 (C9), 37.3 (C10), 21.0 (C11), 27.2 (C12), 37.8 (C13), 43.2 (C14), 27.5 (C15), 35.5 (C16), 43.2 (C17), 48.8 (C18), 43.6 (C19), 41.9 (C20), 23.9 (C21), 39.8 (C22), 28.1 (C23), 15.5 (C24), 16.2 (C25), 16.2 (C26), 14.6 (C27), 17.9 (C28), 179.9 (C29), 17.4 (C30). **IR (KBr) *ν*/cm^−1^**: 3,556; 3,478; 3,416; 3,238; 2,942; 1,638; 1,618; 622; 484. **HR‐H‐ESI‐MS** *m/z* 457.36872 [M]^−^ (calcd for C_30_H_49_O_3_ 457.37)

## 3 Results and Discussion

Our attempts to follow the Principles of Green Chemistry [20] let us initiate our catalytic studies using the best manganese porphyrin studied in our previous lupeol oxidation [19], [Mn(III)(T4CMPP)Cl], a greener solvent (ethyl acetate), and a less dangerous oxidant, PhI(OAc)_2_. While recognizing that the atomic economy of this oxidant may not be optimal, its choice represents a significant step toward making the system greener. PhI(OAc)_2_ offers reduced toxicity and improved handling characteristics compared to iodosylbenzene (PhIO), previously used in similar systems, which is highly toxic, can form an explosive polymer, is insoluble in most organic solvents, and is commercially unavailable [24]. In this way, this choice of oxidant aligns with green chemistry principles to prevent pollution and design safer chemicals. Although hydrogen peroxide (H_2_O_2_) is widely recognized as a green oxidant, related reports on the [Mn(III)(T4CMPP)Cl] catalyst associated to hydrogen peroxide [25, 26, 27] achieved lower yields than iodoarenes derivatives and usually required a co‑catalyst to achieve efficient oxidation. To avoid adding variables and to ensure robust performance across substrates, we employed PhI(OAc)_2_ in this study. A comprehensive evaluation of H_2_O_2_ activation strategies will be the subject of future work. In addition, the catalytic reaction system, which employs ethyl acetate as a harmless solvent and operates at a mild temperature of 50°C, indicates a significant shift towards a safer and more eco‐friendly approach in obtaining lupeol derivatives. This is especially important when compared to traditional methods, which are frequently dangerous for achieving similar transformations.

**FIGURE 1:**
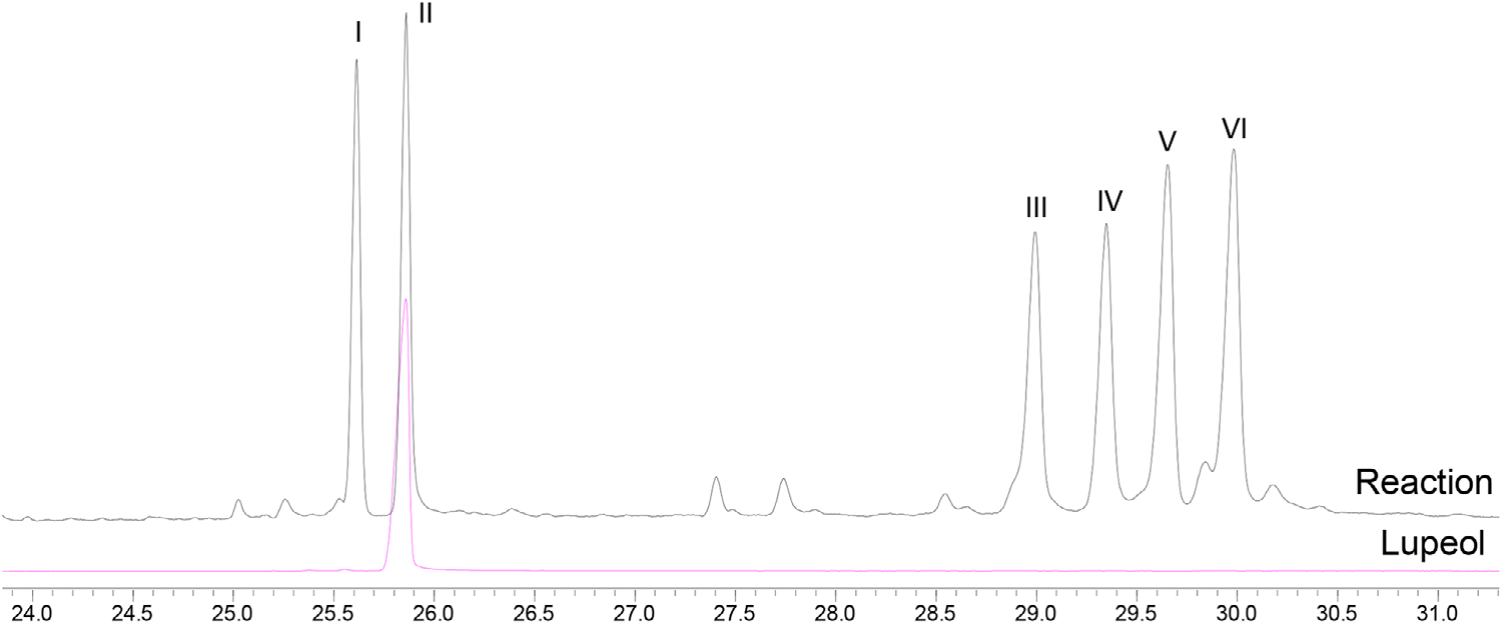
GC profile of product mixture after lupeol oxidation by [Mn(III)(T4CMPP)Cl] + PhI(OAc)_2_ and comparison with lupeol standard.

The first experiment was performed using ethyl acetate, lupeol (0.33 mmol), Mn‐porphyrin (0.0099 mmol), and PhI(OAc)_2_ (0.40 mmol), with a molar ratio MnP:lupeol:PhI(OAc)_2_ equal to 1:33:40 (the same as previously used) [19]. The reaction mixture, under greener conditions, was magnetically stirred for 4 h at 0°C. Unfortunately, lupeol remained unreacted, and no product was observed by gas chromatography or TLC analysis. Based on this result, temperature, oxidant quantity, and the reaction time were increased in a new experiment. The new system was carried out under ethyl acetate, using lupeol (0.33 mmol), Mn‐porphyrin (0.0099 mmol), and PhI(OAc)_2_ (0.99 mmol), with a molar ratio MnP:lupeol:PhI(OAc)_2_ equal to 1:33:100. The reaction mixture was magnetically stirred for 24 h at 50°C. Analysis of the gas chromatograms (Figure 1) revealed the formation of five products (peaks I, III, IV, V, and VI) and the presence of unreacted lupeol (peak II), identified by comparison with an authentic sample. Although GC‐MS analysis showed a high conversion of lupeol (94%), the isolated yield of the characterized products was 11%. This difference can be associated with the intrinsic challenges in isolating and quantifying all compounds from the complex mixture (eight products, catalyst, etc.), mainly those present in low concentrations, as well as probable losses during the purification, realized in a multi‐step process. The GC conversion represents the total consumption of the starting lupeol, while the reported yields are associated with the purified and characterized products.

**FIGURE 2:**
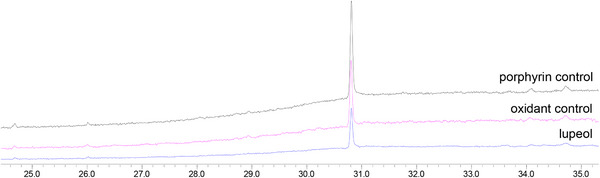
GC profile comparison between the two control reactions and lupeol.

Control reactions [22, 28, 29] (Figure 2), in the absence of oxidant (porphyrin control) or catalyst (oxidant control), were carried out under the same conditions as the catalytic runs. Comparison between the GC profiles of the two controls and the lupeol standard (Figure 2) showed the absence of any product and the exclusive presence of lupeol. This result was confirmed by a TLC analysis. These findings highlighted the importance of the combination of porphyrin and oxidant to perform the oxidation of lupeol.

**FIGURE 3:**
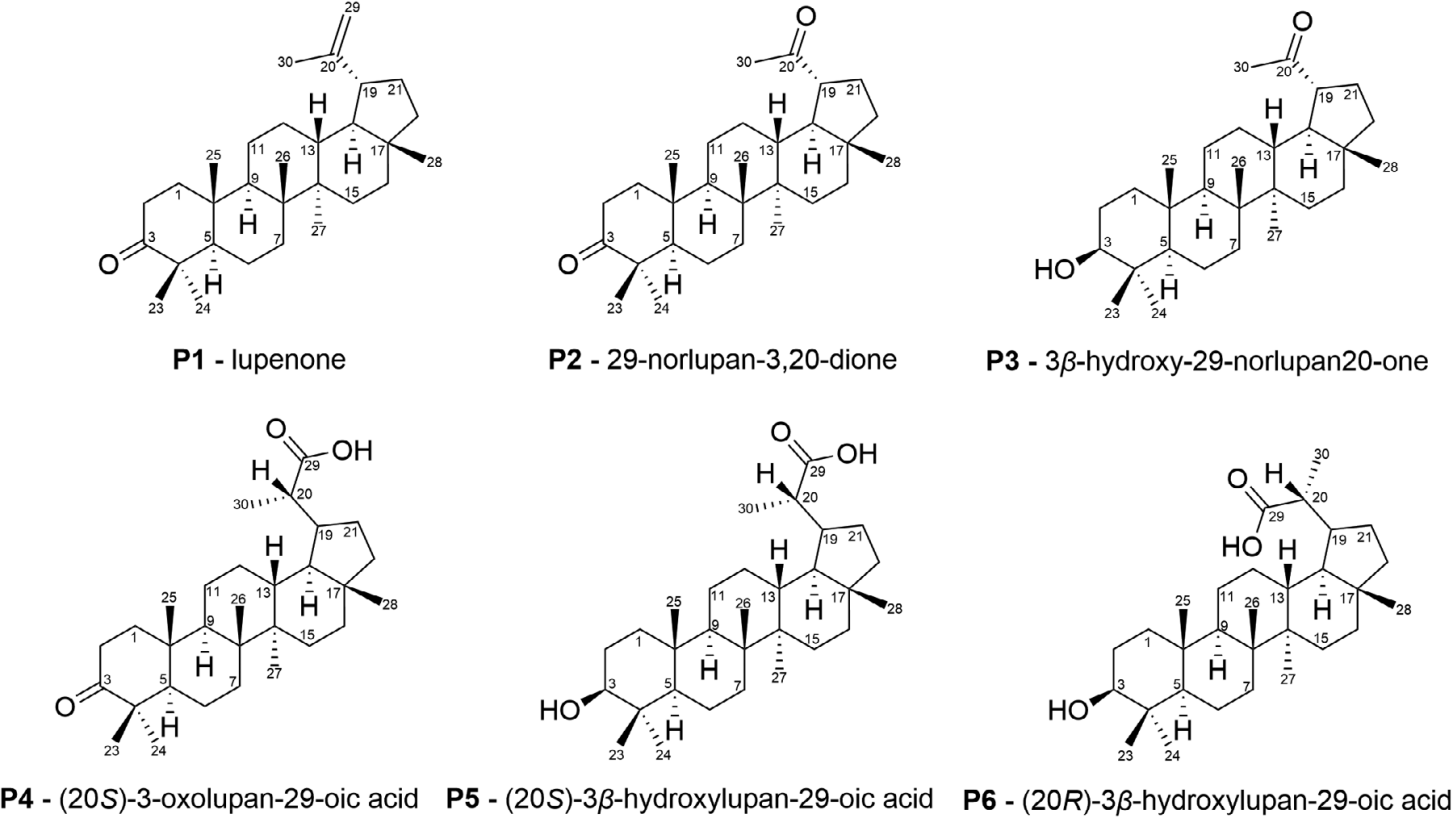
Products obtained after lupeol oxidation by [Mn(III)(T4CMPP)Cl]+PhI(OAc)_2_.

Given these results, the reaction was performed in several replicates (23) to isolate and characterize each of the products. The combination of these reaction mixtures was treated through column chromatography and preparative layer chromatography (PLC) for product isolation. In total, six products (Figure 3) were isolated and successfully characterized by ^1^H and ^13^C NMR and IR (Figures S1‐S22). The structures of products **P1‐P4** were determined by comparison of the ^13^C NMR with the literature data [19, 30, 31, 32]. As no ^13^C NMR data were found in literature for diastereoisomers **P5** and **P6**, structural determination was carried out by comparison with data from similar molecules [30]. The configurations at C20 for **P4**, **P5**, and **P6** were assigned based on comparison of their ^13^C NMR data with literature precedents for these specific stereoisomers. It is important to highlight that, given the achiral nature of the catalytic conditions, the formation of a mixture of diastereomers is expected for chiral products. The ^13^C NMR signals for both diastereoisomeric products **P5** and **P6** are herein described for the first time (Figures S17 and S21).

**FIGURE 4:**
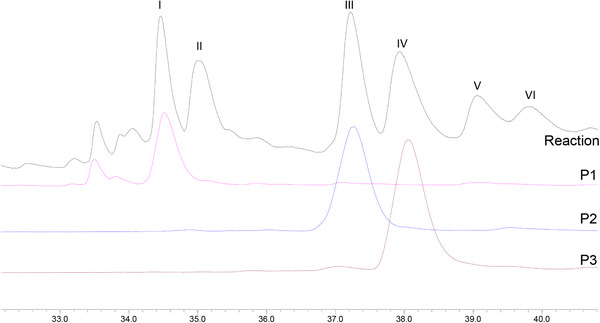
GC profile comparison of product mixture after lupeol oxidation by [Mn(III)(T4CMPP)Cl] + PhI(OAc)_2_ and isolated products P1, P2, and P3.

Through comparison of the GC profiles and mass spectra of the isolated compounds with those of the reaction mixture (Figure 4), peaks I, III, and IV were identified as corresponding to products **P1**, **P2**, and **P3**, respectively. Due to their low volatility, products **P4**, **P5**, and **P6** were not analyzed by GC‐MS. Products from peaks V and VI could not be isolated after purification by column chromatography.

Lupenone (**P1**) has been isolated from several plant species [17, 31, 33, 34, 35, 36, 37, 38, 39] and synthesized from lupeol via PCC oxidation [40] or Jones oxidation [41]. **P1** displays a vast range of biological activities, such as antidiabetic effects, anti‐inflammatory, antiviral, anticancer, and anti‐Chagas disease [42]. 29‐norlupan‐3,20‐dione (**P2**) has been isolated from plants [34, 38, 43, 44] and synthesized from lupeol in two manners: using OsO_4_ and NaIO_4_, with subsequent Dess‐Martin oxidation [44] or using Oxone followed by K_2_Cr_2_O_7_/H_2_SO_4_ oxidation [45]. **P2** displays antimicrobial activity against *M. miehi* and *C*. *albicans* [34] and antitoxoplasma activity [44]. 3*β*‐hydroxy‐29‐norlupan‐20‐one (**P3**) has been isolated from several plants [17, 18, 33, 36, 37, 38, 39, 43, 46, 47, 48, 49, 50, 51, 52, 53, 54] and synthesized from lupeol in two manners: OsO_4_ addition with subsequent cleavage of the diol with NaIO_4_ [44] or direct oxidation of lupeol with Oxone in acetic acid [45, 55]. **P3** was also obtained via oxidation by iodosylbenzene mediated by manganese porphyrin [19] and, to the best of our knowledge, no further works using porphyrins to oxidize lupeol to **P3** have been published. **P3** exhibits antileishmanial, antitrypanosomal [18] and antioxidant [16] activity and reduces NO production in BV‐2 cells [17]. (20*S*)‐3‐oxolupan‐29‐oic acid (**P4**) has been isolated from *Euonymus* *carnosus* [30], but no synthesis from lupeol has been reported in the literature. Zhou et al. [30] have investigated **P4** as a potential anticancer agent against several cancer lineages and its ability to induce the formation of NO in BV2 cells, but **P4** did not display any activity in both cases. (20*S*)‐3*β*‐hydroxylupan‐29‐oic acid (**P5**) and (20*R*)‐3*β*‐hydroxylupan‐29‐oic acid (**P6**) have been isolated from plants [46, 49, 56, 57, 58] and synthesized from lupenyl acetate in a lengthy 4‐step racemic synthesis [59]. No biological activities have been reported for these products. It is important to highlight that our method provides a concise route to these known biologically relevant compounds, that are **P1**, **P2**, and **P3**.

Conversion of a terminal alkene into a carboxylic acid, as observed in this study to form **P4**, **P5**, and **P6** from lupeol, can be achieved either by a one‐pot ozonolysis‐oxidation process [60] or a two‐step protocol involving hydroxylation to form a 1,2‐diol followed by a periodic acid (HIO_4_) cleavage [60, 61]. The one‑pot ozonolysis–oxidation of terminal alkenes has been explicitly documented only once, for a variety of primary alkenes [60] and, to the best of our knowledge, has not previously been applied to lupeol. Ozonolysis is accurate, but the chemical process requires a comprehensive approach that includes risk assessments and safety protocols, as ozone gas is highly toxic, can form explosive ozonides with many alkenes, and its generation requires special instrumentation. Periodic acid is considered a hazardous substance, according to the US Occupational Safety and Health Administration [62]. In contrast, the use of [Mn(III)(T4CMPP)Cl] as the catalyst for the PhI(OAc)_2_‐oxidation of lupeol to yield products **P4**, **P5**, and **P6** represents a groundbreaking route for the transformation of the alkene into a carboxylic acid derivative of this lupane under milder and greener conditions, avoiding the hazards associated with conventional methods.

To the best of our knowledge, the present work is the first example of this specific reaction (carboxylic acid from alkene) on lupeol by metalloporphyrin‐based catalysis or any other oxidative process under controlled and less hazardous conditions. The oxidations of lupeol using conventional methods are already described in the literature and they presented C20 modifications on lupeol mainly as epoxidation [41, 63], direct allylic alcohol formation [14, 19, 64], formylation [13, 14, 15, 41, 64, 65, 66], C20 = C29 bond cleavage (with loss of the methylene carbon C29) to yield a ketone [19, 45], and successive oxidations in its isopropenyl side chain, followed by successive decarboxylation [11].

**FIGURE 5:**
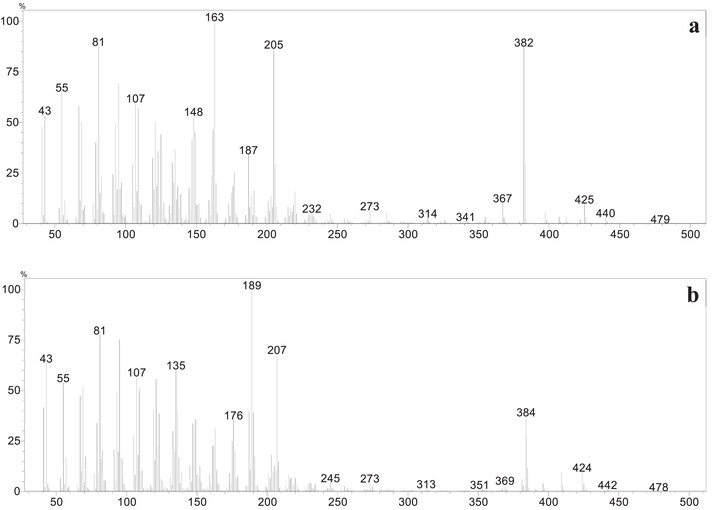
(a) Mass spectra of peak V in the GC profile of the product mixture; (b) mass spectra of peak VI in the GC profile of the product mixture.

**SCHEME 2:**
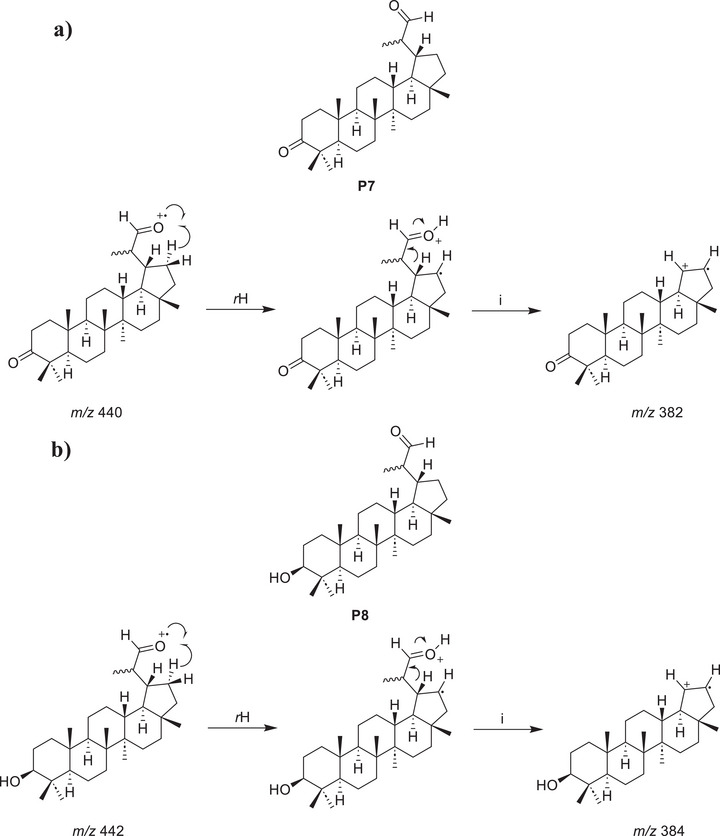
(a) Proposed structural assignment for the probable aldehyde (P7) corresponding to peak V and proposed fragmentation pathway leading to the formation of the *m/z* 382; (b) proposed structural assignment for the probable aldehyde (P8) corresponding to peak VI and proposed fragmentation pathway leading to the formation of the *m*/*z* 384.

During the purification process, no correspondence was observed for the peaks V and VI in the reaction mixture chromatogram (Figure 4) and the isolated products **P4**, **P5**, and **P6**. However, analysis of the mass spectra corresponding to each peak allows the proposition of plausible structures for these products, named **P7** and **P8**. In the mass spectrum corresponding to peak V (Figure 5a), a possible molecular ion peak was observed at *m/z* 440, along with an intense peak at *m/z* 382. Based on the chemical structures of products **P4**, **P5,** and **P6**, which contain carboxyl groups, and the characteristic loss of 58 atomic mass unities (McLafferty rearrangement) from the molecular ion, it is strongly proposed that chromatographic peak V may correspond to an aldehyde **P7** (Scheme 2a). Similarly, for peak VI (Figure 5b), the mass spectrum of peak VI showed a possible molecular ion peak at *m/z* 442 and a peak at *m/z* 384, corresponding to a loss of 58 atomic mass unities, the same loss observed in the mass spectra of peak V, consistent with the proposal of an analogue aldehyde (**P8**) presenting a hydroxyl group at C3 and undergoing a similar fragmentation. The fragmentation of the molecular ion at *m/z* 442 leading to the peak at *m/z* 384 would also result from a McLafferty‐like rearrangement, as represented in Scheme 2b.

While these mass data strongly supported the presence of aldehydes, to corroborate the proposal for these aldehydes in the reaction mixture, Fehling's test was performed [67]. A few drops of the reaction mixture were added to a test tube containing an aqueous solution of copper (II) sulfate and sodium potassium tartrate (Fehling's solution). Upon heating, the color of the solution changed from deep blue to light green, and a brownish‐orange precipitate was formed at the bottom of the tube, indicating a positive result for aldehydes. Considering the inherent instability and low concentration of these proposed aldehydes (**P7** and **P8**), it was not possible to perform their complete isolation and characterization. It is important to highlight that the lack of isolation and full spectroscopic characterization of **P7** and **P8** precludes their unambiguous structural proof currently. The structural proposition relies on classical qualitative tests and expected reactivity trends, and the definitive structures await isolation and full analyses (e.g., NMR, MS, IR, and, if possible, x‑ray). The presented analysis on **P7** and **P8** proposed structures may encourage pursuing isolation and definitive characterization in future studies, which could confirm or revise the proposed structures.

To verify if the isolated carboxylic acids (**P4**, **P5,** and **P6**) were formed due to the spontaneous oxidation of the proposed aldehydes (**P7** and **P8**) or if they were also formed as products, before the chromatographic isolation, a few drops of the reaction mixture were added to a test tube, followed by the addition of solid sodium bicarbonate. The evolution of bubbles indicated the presence of carboxylic acids, suggesting that these products are also formed in the reaction media. A control test, performed only in the presence of ethyl acetate (the same solvent used in the catalyzed reaction), didn't result in the formation of bubbles, indicating that a false positive result didn't occur using the reaction media.

**P7**, as proposed, has been previously isolated from plants [68, 69, 70] and synthesized from lupenone (**P1**) via ozonation [71], but no synthesis from lupeol has been published until today. This proposed product **P7** displays antimicrobial activity against *Staphylococcus aureus* [72]. **P8**, as proposed, has also been isolated from plants [37, 46, 68]; notably, Mutai et al. [68] were able to isolate only the *S* epimer from *Acacia mellifera*. **P8**, considering the proposed structure, exhibits antioxidant activity [16].

**FIGURE 6:**
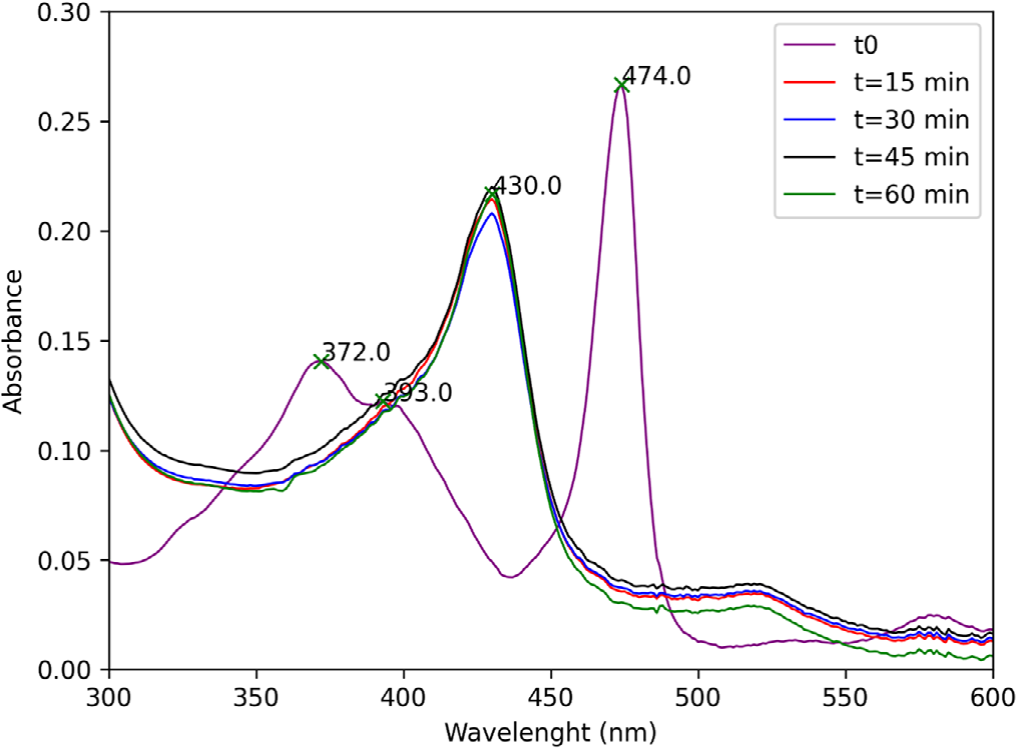
Time course of the lupeol oxidation catalyzed by [Mn(III)(T4CMPP)Cl] as monitored by UV–vis (ethyl acetate): (a) initial spectrum of [Mn(III)(T4CMPP)Cl]; (b) [Mn(III)(T4CMPP)Cl] + PhI(OAc)_2_ (15 min of reaction); (c) [Mn(III)(T4CMPP)Cl] + PhI(OAc)_2_ (30 min of reaction); (d) [Mn(III)(T4CMPP)Cl] + PhI(OAc)_2_ (60 min of reaction).

Monitoring the catalytic runs by UV–vis spectroscopy revealed that, upon addition of PhI(OAc)_2_ to the [Mn(III)(T4CMPP)Cl]+lupeol systems, the initial Mn(III) porphyrin species has its color changed from green to brown, which means the catalyst is completely converted to a new species, which has the *Soret* band shifted to the blue by 49 nm with respect to the *Soret* of the initial Mn(III) complex (474 nm to 430 nm) (Figure 6). This new species is quite stable and persists as the major observable species throughout the entire run. This observed species is likely a Mn(IV)‐oxo, as already seen in a previous study for [Mn(III)(T4CMPP)Cl]+PhIO [22]. It is important to highlight that while Mn(IV)‐oxo species represents a stable intermediate, the highly reactive and short‐lived Mn(V)‐oxo are generally accepted as the catalytic oxidant in such biomimetic systems. Due to their fast turnover, direct detection of Mn(V)‐oxo species remains a challenge under typical experimental conditions used [28, 29], aiding in the identification of Mn(IV)‐oxo as the main species detectable through spectroscopy.

Although it has been known that the association of manganese(III) porphyrin complexes with oxidants, such as hypervalent iodoarenes, lead to the formation of high‐valent manganese(V)‐oxo porphyrins ([MnV(O)P]) [73, 74, 75], responsible for the transformation of the substract, we performed some experiments to verify if another mechanistic pathway could be responsible for the oxidation of lupeol, involving or not involving the [MnV(O)P] species. We carried out the same reaction with mannitol (**I1**), a known inhibitor of hydroxyl (·OH) radicals [76], sodium azide (NaN_3_, **I2**), a singlet oxygen (^1^O_2_) trap [77], and butylated hydroxytoluene (BHT, **I3**), which can inhibit several radical species, including organic radicals derived from the oxidant PhI(OAc)_2_ [78]. All radical scavengers were tested individually in the same molar ratio as the oxidant, and analyzed by GC‐MS. The results are presented in Figure S23.

The effect of some radical scavengers on the reaction was investigated to obtain preliminary insights into the mechanistic pathways. The presence of mannitol and NaN_3_ completely inhibited the formation of **I**, **III**, and **IV**, suggesting the potential involvement of singlet oxygen and hydroxyl radicals in the products formation. In the presence of BHT, only lupeol was observed, hinting that this scavenger might interfere with broader radical pathways, possibly including the formation of the [MnV(O)P] species, which was typically implicated in the formation of various oxidized products. Although these scavenger tests offer promising insights, additional conclusive mechanistic studies are necessary to clarify the intricate interactions of pathways, such as radical generation, singlet oxygen, and the role of metal‐oxo compounds.

It is important to highlight that the change in conditions likely impacts the accessibility or reactivity of the C3‐hydroxyl group of lupeol, making it susceptible to the P450‐mimetic system, which might not have been a dominant pathway under the conditions of a previous work by Martins et al. [19]. The formation of carboxylic acids (**P4**‐**P6**) from the terminal alkene or its preliminary oxidation derivatives (for instance, proposed aldehydes **P7**/**P8**) suggests a more intense oxidative environment. The unique combination of oxidant, solvent, and temperature used in this research seems to promote or enable the continued oxidation of intermediate aldehydes to carboxylic acids, a reaction that might have been less common or entirely inhibited in our previous experimental context [19]. It is known that porphyrin‐based catalysts can exhibit highly variable reactivity and selectivity under different environmental or reaction conditions, acting as chameleons in cytochrome P450 model reactions [79]. In the present study, the observed product distribution serves as strong evidence for the adaptable nature of these porphyrin‐based catalysts.

The degree of the destruction of the [Mn(III)(T4CMPP)Cl] was determined at the end of the catalytic runs by diluting an aliquot of the reaction mixture in ethyl acetate and measuring the UV–vis; the recovery was calculated based on the molar absorptivity of the *Soret* band of the Mn(III) porphyrin complex. The partial catalyst decomposition (87%) did not limit lupeol conversion.

## 4 Conclusion

The PhI(OAc)_2_‐oxidation of lupeol, catalyzed by a Mn‐porphyrin as a Cytochrome P450‐based metabolism reaction model, was investigated. A high lupeol conversion was achieved (94%), and eight products were formed; for some of them, this is the first report of these products being obtained from lupeol. Six of these products were properly isolated and characterized, while the other two had their structures proposed based on chemical tests and known reactivity of organic molecules. The strategic combination of more benign reaction conditions, including ethyl acetate as solvent and iodobenzene diacetate as a safer alternative to conventional oxidants, coupled with the [Mn(III)(T4CMPP)Cl] catalyst, establishes a novel route to semi‐synthetic derivatives of lupeol. This method eliminates the need for prior protection of the C3 hydroxyl group of lupeol, a significant shortening. Although lupeol is considerably resistant to oxidative transformations (and possibly of difficult metabolism), through our biomimetic approach it was possible to successfully achieve eight distinct products for which it would be necessary the use of more hazardous materials and rough conditions. This work presented the selective C20 functionalization of lupeol without the need for protection of its C3‑OH group using a safer oxidant system; establishing generality across diverse substrates under unified conditions is the subject of ongoing studies. The present study reinforces the considerable potential use of porphyrins and their derivatives as catalysts for the transformation of other natural products of biological interest, providing a route to diverse frameworks important for drug development.

## Author Contributions

**Leila Renan Oliveira** performed the synthesis, product purification, spectroscopic analysis, GC‐MS analysis, wrote the first draft of the manuscript, and analyzed the data. **Pedro Fonseca‐Pinheiro** performed catalytic reactions, monitored the porphyrin decomposition, and wrote the first draft of the manuscript. **Lucienir Pains Duarte** contributed to study conceptualization, experimental design, formal analyses, and commented on previous versions of the manuscript. **Diogo Montes Vidal** contributed to study conceptualization, experimental design, formal analyses, and commented on previous versions of the manuscript. **Grasiely Faria de Sousa** contributed to study conceptualization, experimental design, formal analyses, and commented on previous versions of the manuscript. **Dayse Carvalho da Silva Martins** contributed to study conceptualization, experimental design, formal analyses, commented on previous versions of the manuscript, and wrote the first draft of the manuscript. All authors read, discussed, and approved the final version of the manuscript.

## Conflicts of Interest

The authors declare no conflicts of interest.

## Associated Contents

The IR and NMR spectra of compounds P1‐P6 and the HR‐H‐ESI‐MS analysis of compounds P5 and P6, are available in supplementary information (Figures S1‐S22), as well as the GC profile comparison of the reaction mixture with the inhibited reactions (Figure S23), and the retention indexes for the compounds P1‐P3, P7‐P8 and lupeol (Table S1).

## Supporting information




**Supporting File 1**: cbdv71239‐sup‐0001‐SuppMat.pdf

## Data Availability

The data that support the findings of this study are available from the corresponding author upon reasonable request.
