# Supplementary material for: Manganese Porphyrin‐Catalyzed One‐Pot Oxidation of Lupeol: Efficient Conversion of Its Terminal Alkene Into Carboxylic Acids
Source: Chem Biodivers. 2026 May 4;23:e71239. doi: 10.1002/cbdv.71239 (PMC13138786; doi:10.1002/cbdv.71239)
Supplement: Supplementary file 1 — Supporting File 1: cbdv71239‐sup‐0001‐SuppMat.pdf [file CBDV-23-e71239-s001.pdf]

Supplementary Information

Manganese Porphyrin-Catalyzed One-pot Oxidation of Lupeol: Efficient Conversion of its Terminal Alkene into Carboxylic Acids

Leila Renan Oliveira[a], Pedro Fonseca-Pinheiro[a], Lucienir Pains Duarte[a], Diogo Montes Vidal[a], Grasiely Faria de Sousa[a], and Dayse Carvalho da Silva Martins[a]*

[a]*Departamento de Química, Instituto de Ciências Exatas, Universidade Federal de Minas Gerais, 31270-901, Belo Horizonte, MG, Brazil*

*daysequimica@ufmg.br

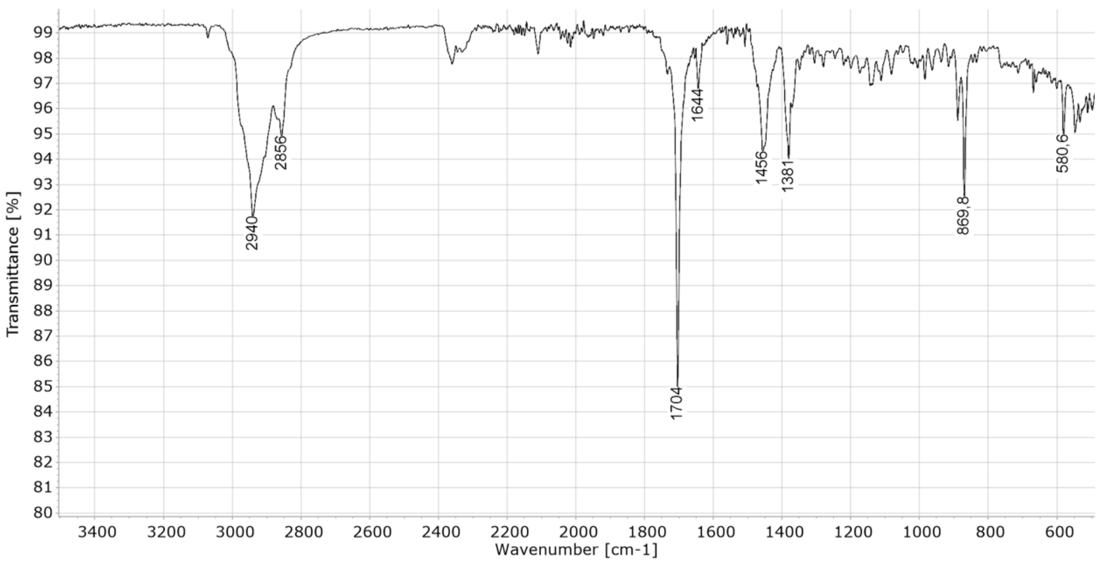

Figure S1. FTIR (ATR) spectrum of **P1**.

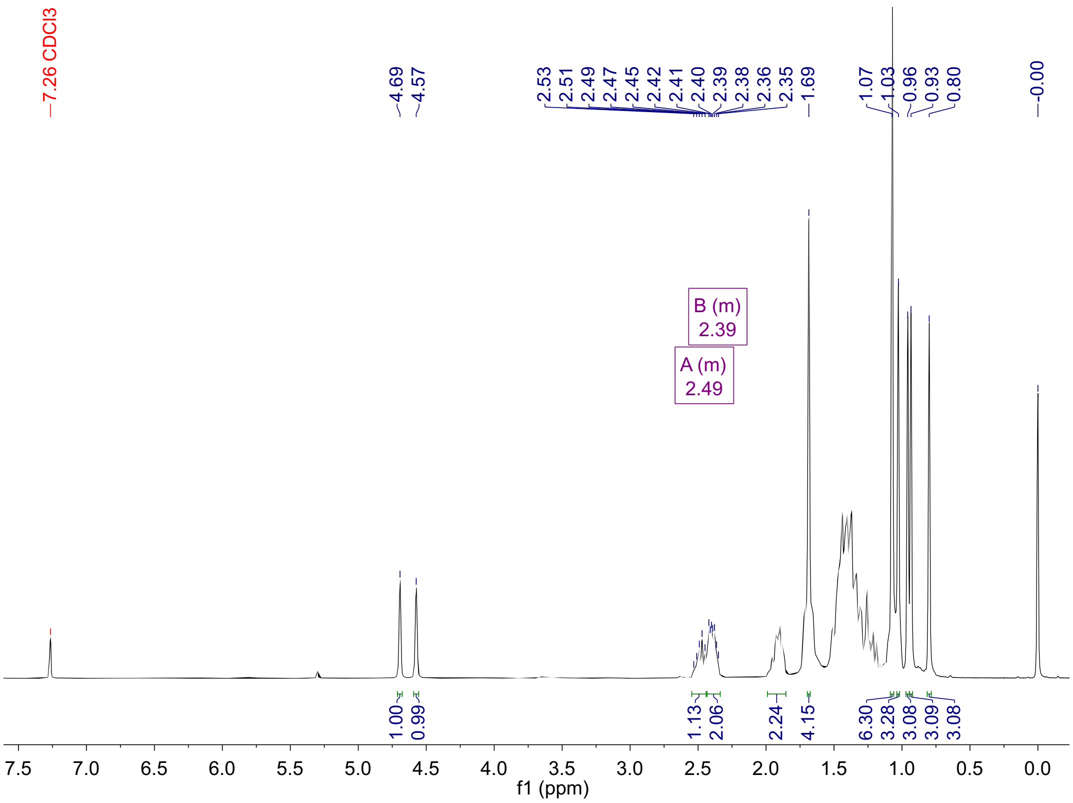

Figure S2. ^{1}H NMR spectrum (400 MHz, $CDCl_3$) of **P1**.

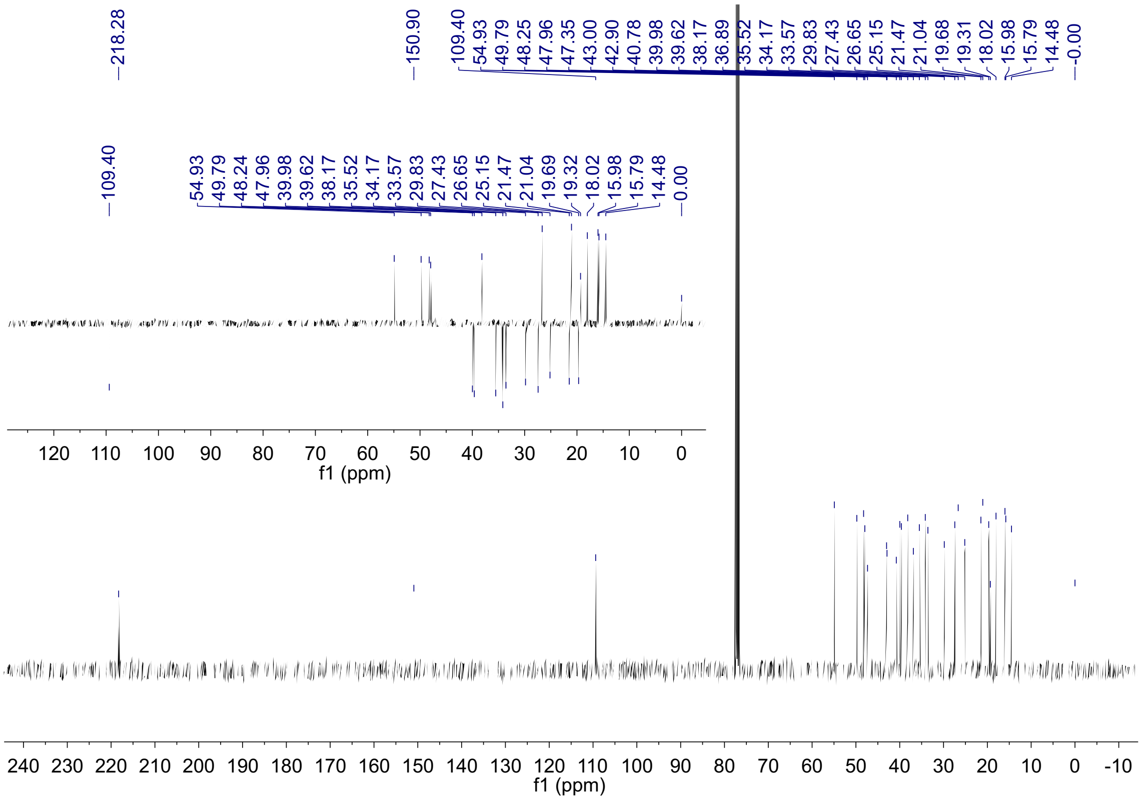

Figure S3. ^{13}C NMR and DEPT-135 spectra (100 MHz, $CDCl_3$) of **P1**.

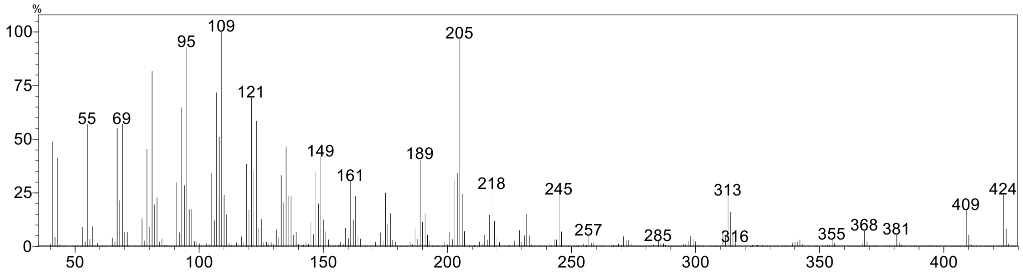

Figure S4. Mass spectrum (EI, 70eV) of **P1**.

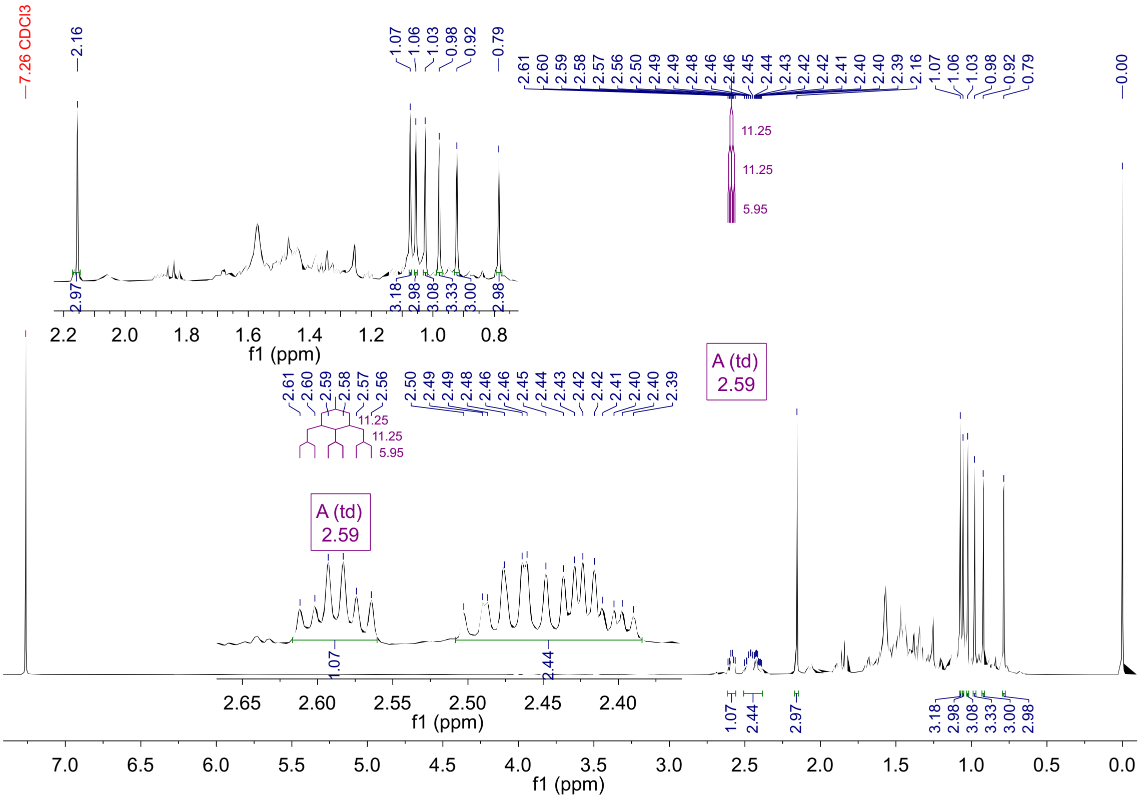

Figure S5. ^{1}H NMR spectrum (600 MHz, $CDCl_3$) of P**2**.

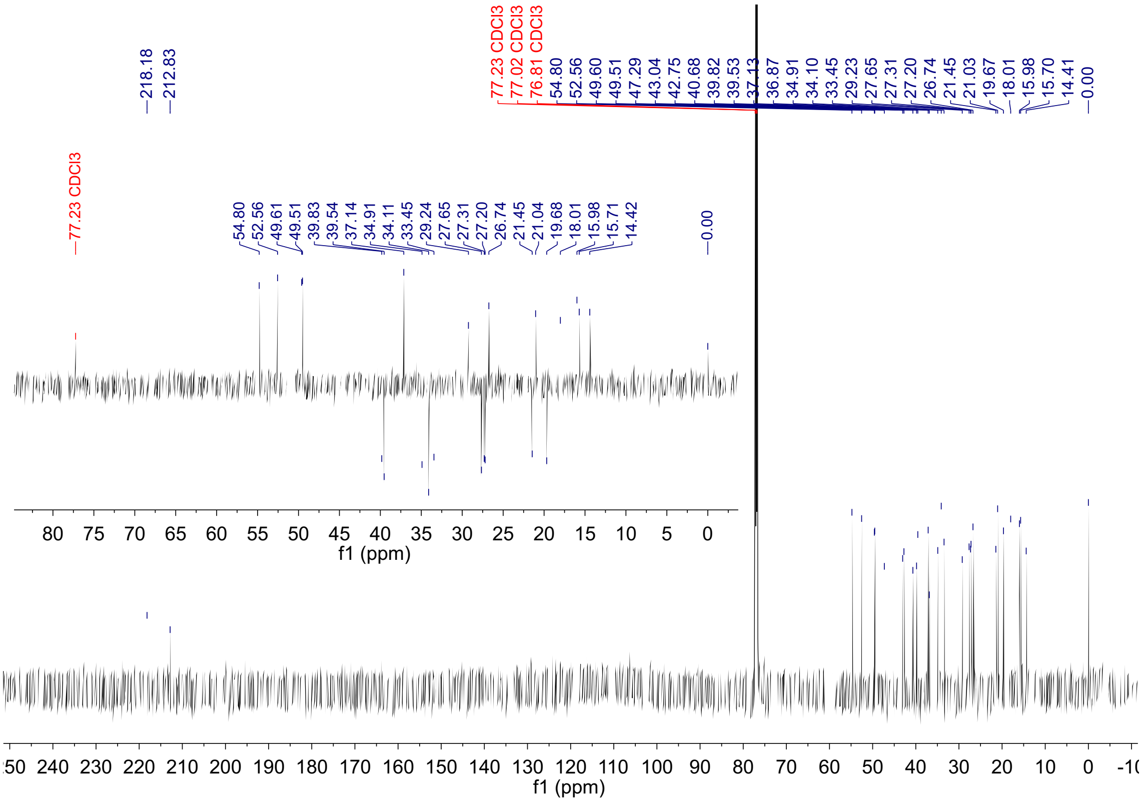

Figure S6. ^{13}C NMR and DEPT-135 spectra (150 MHz, $CDCl_3$) of **P2**.

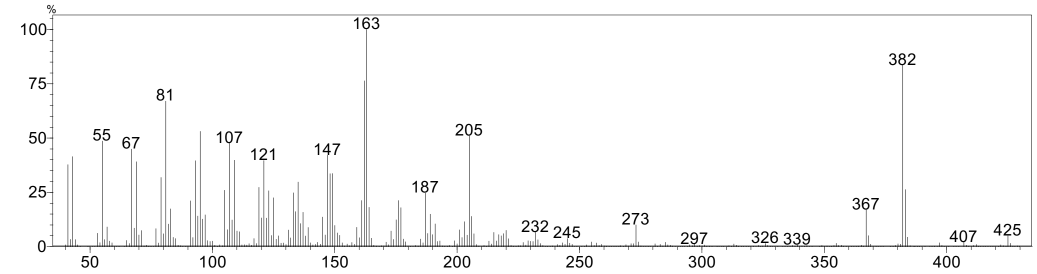

Figure S7. Mass spectrum (EI, 70eV) of **P2**.

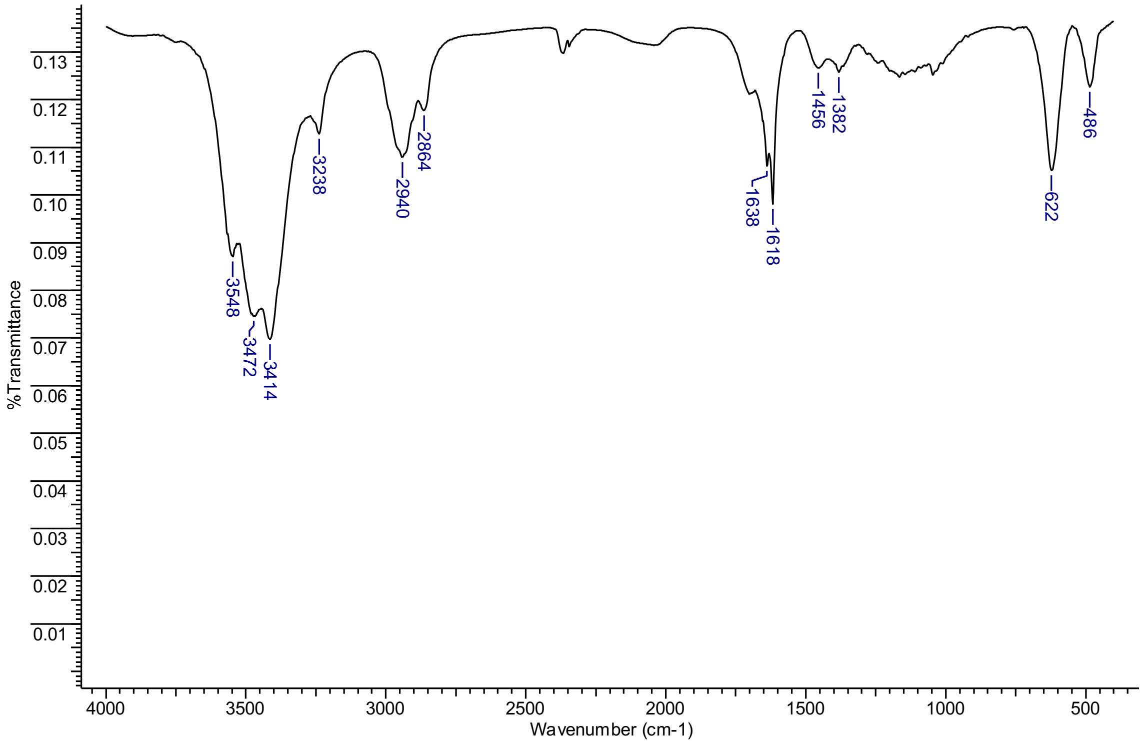

Figure S8. FTIR (KBr) spectrum of **P3**.

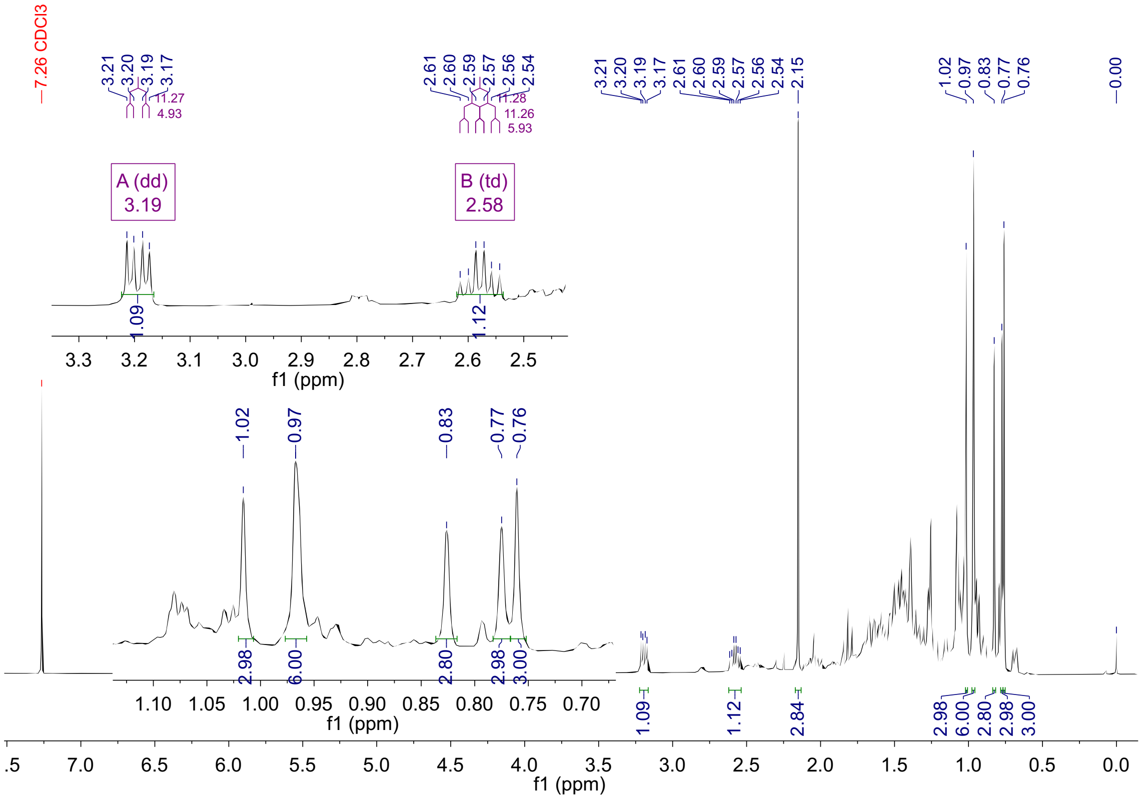

Figure S9. ^{1}H NMR spectrum (400 MHz, $CDCl_3$) of compound **P3**.

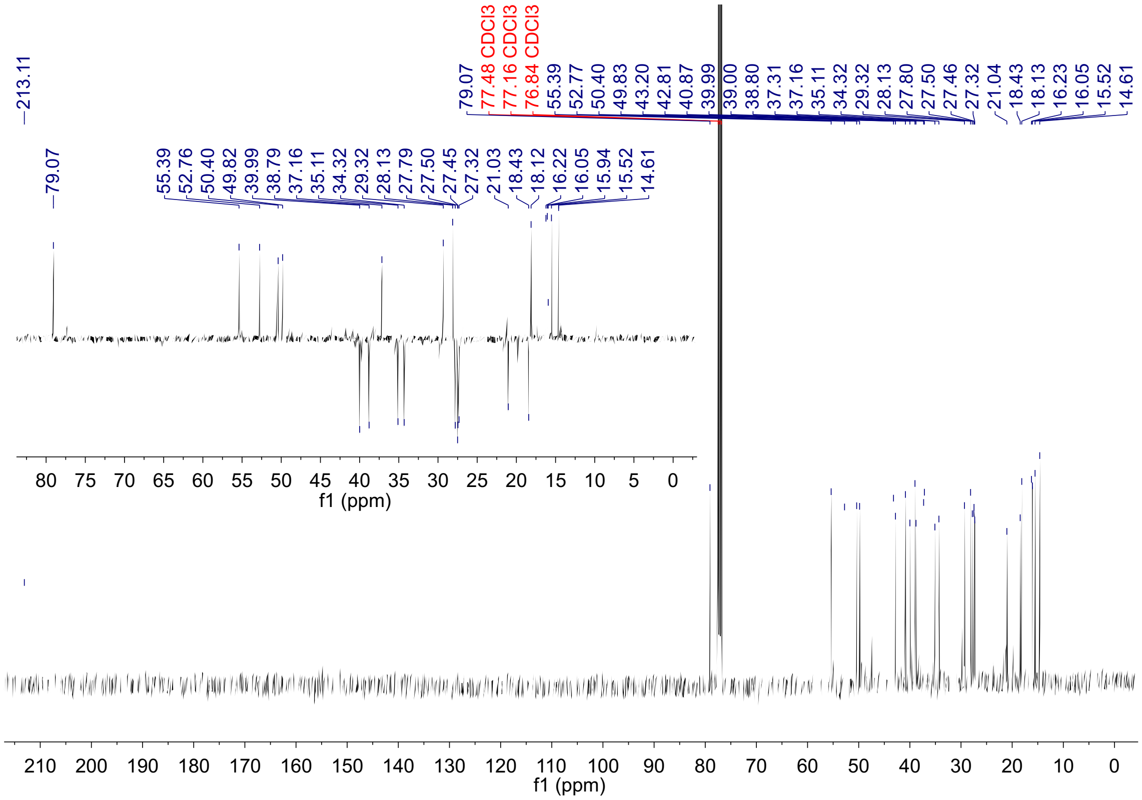

Figure S10. ^{13}C NMR and DEPT-135 spectra (100 MHz, $CDCl_3$) of **P3**.

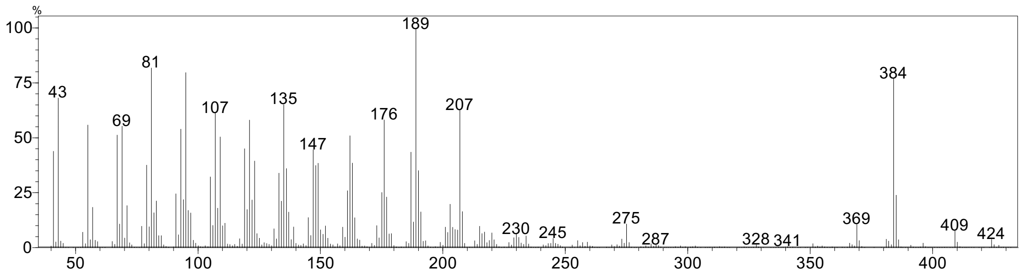

Figure S11. Mass spectrum (EI, 70eV) of **P3**.

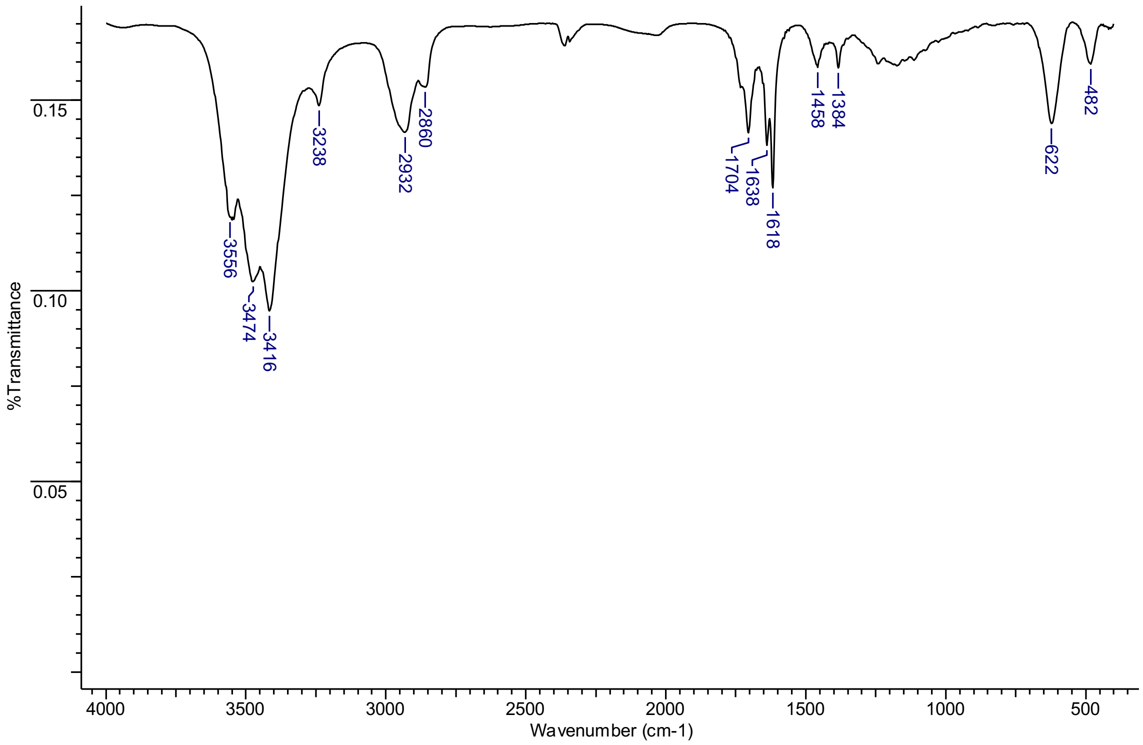

Figure S12. FTIR (KBr) spectrum of **P4**.

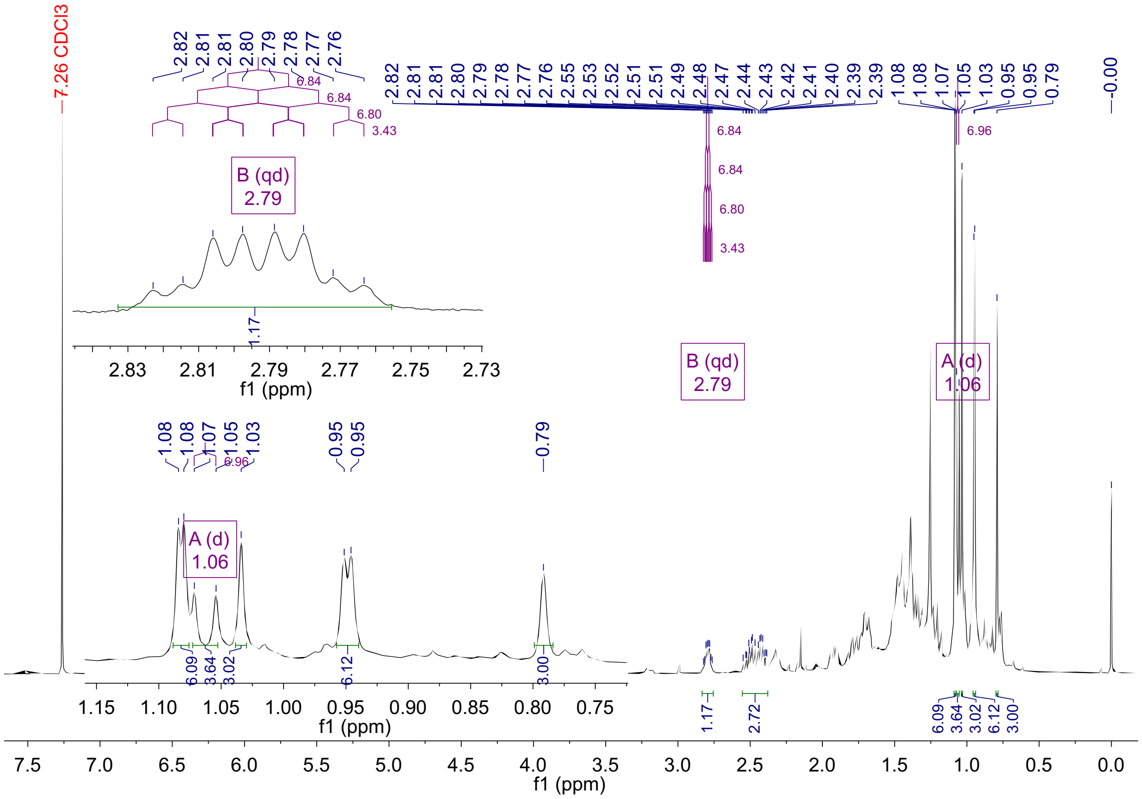

Figure S13. ^{1}H NMR spectrum (400 MHz, $CDCl_3$) of **P4**.

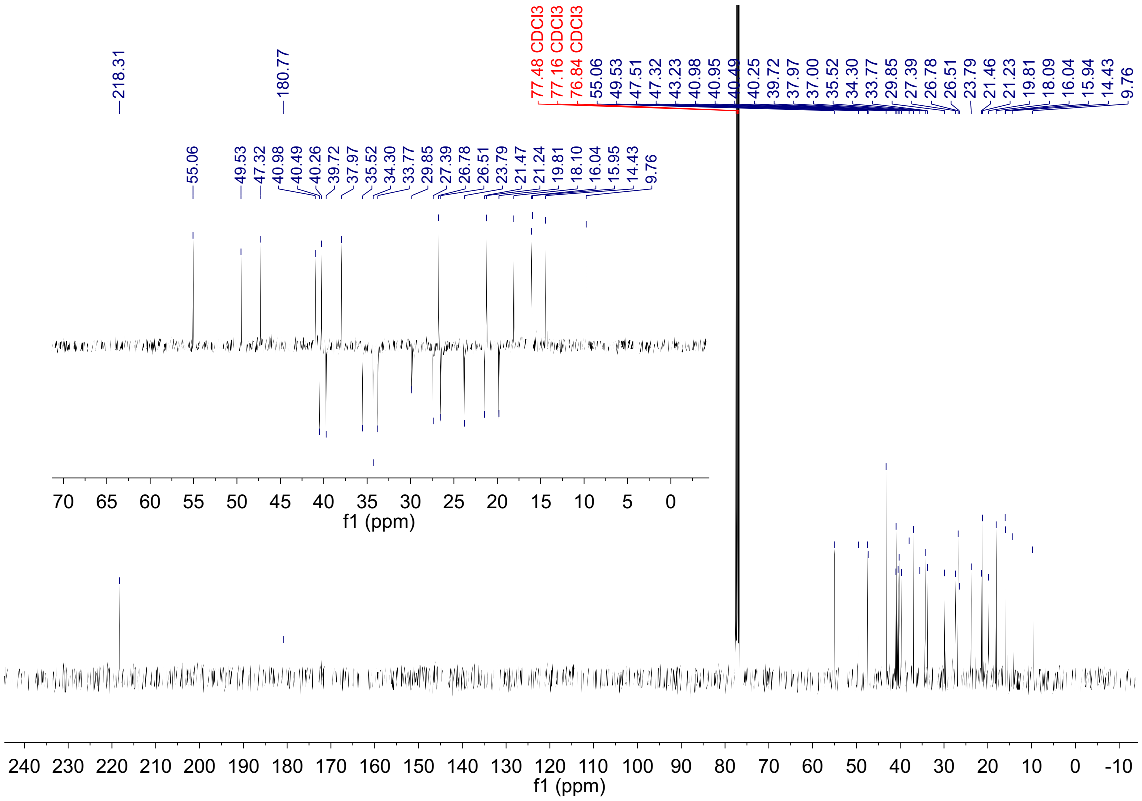

Figure S14. ^{13}C NMR and DEPT-135 spectra (100 MHz, $CDCl_3$) of compound **4**.

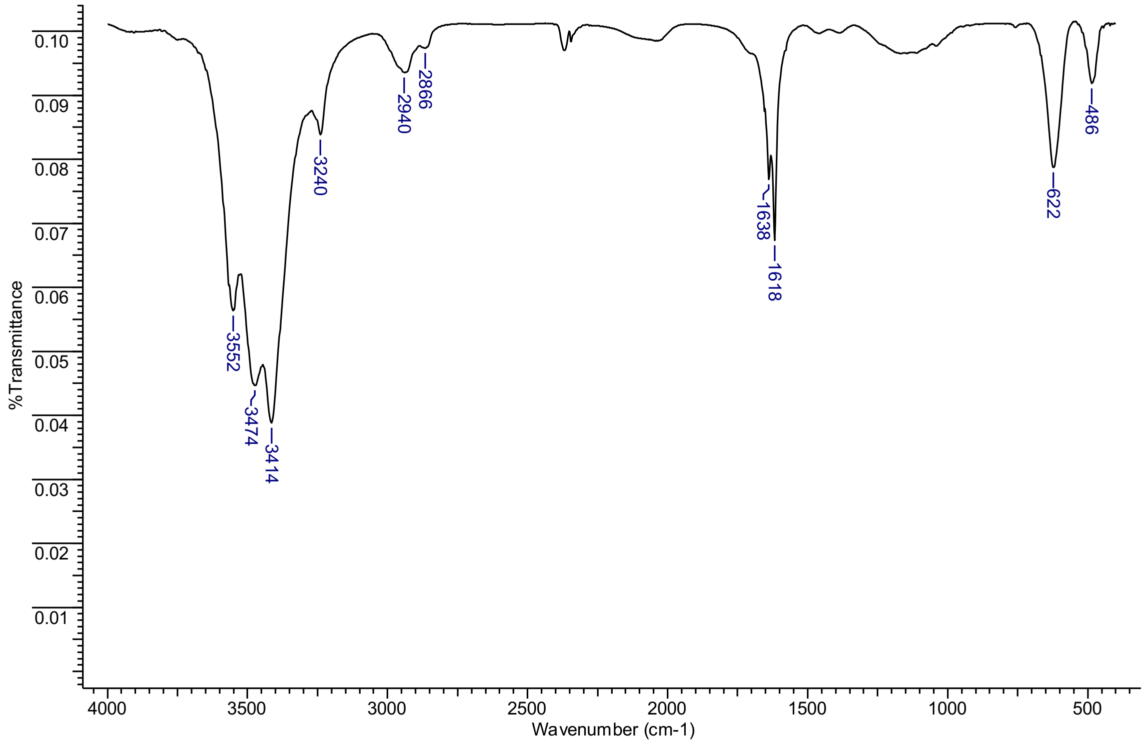

Figure S15. FTIR (KBr) spectrum of **P5**.

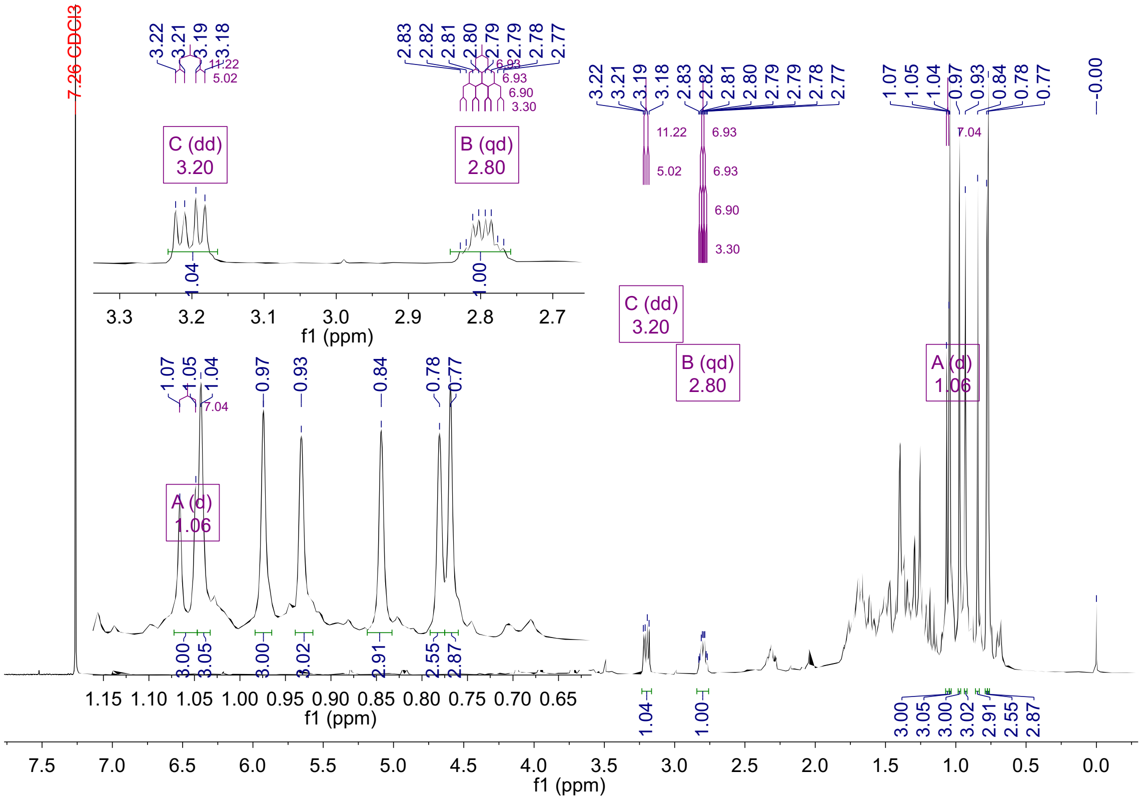

Figure S16. ^{1}H NMR spectrum (400 MHz, $CDCl_3$) of **P5**.

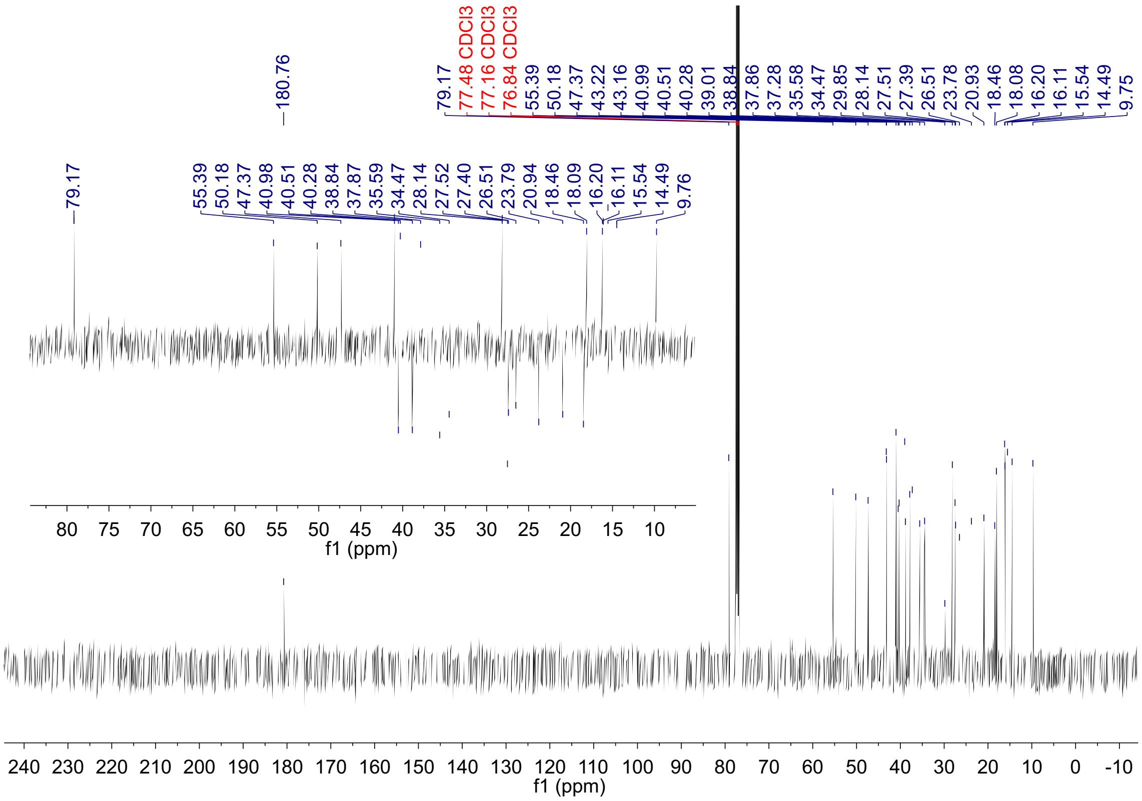

Figure S17. ^{13}C NMR and DEPT-135 spectra (100 MHz, $CDCl_3$) of **P5**.

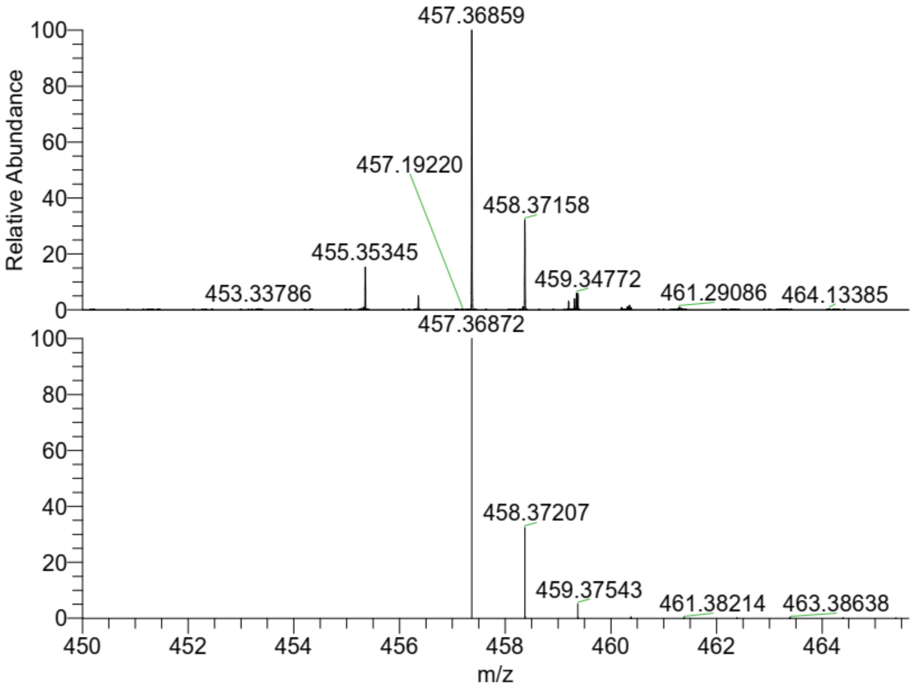

Figure S18. Mass spectrum (HR-H-ESI-MS) of **P5**.

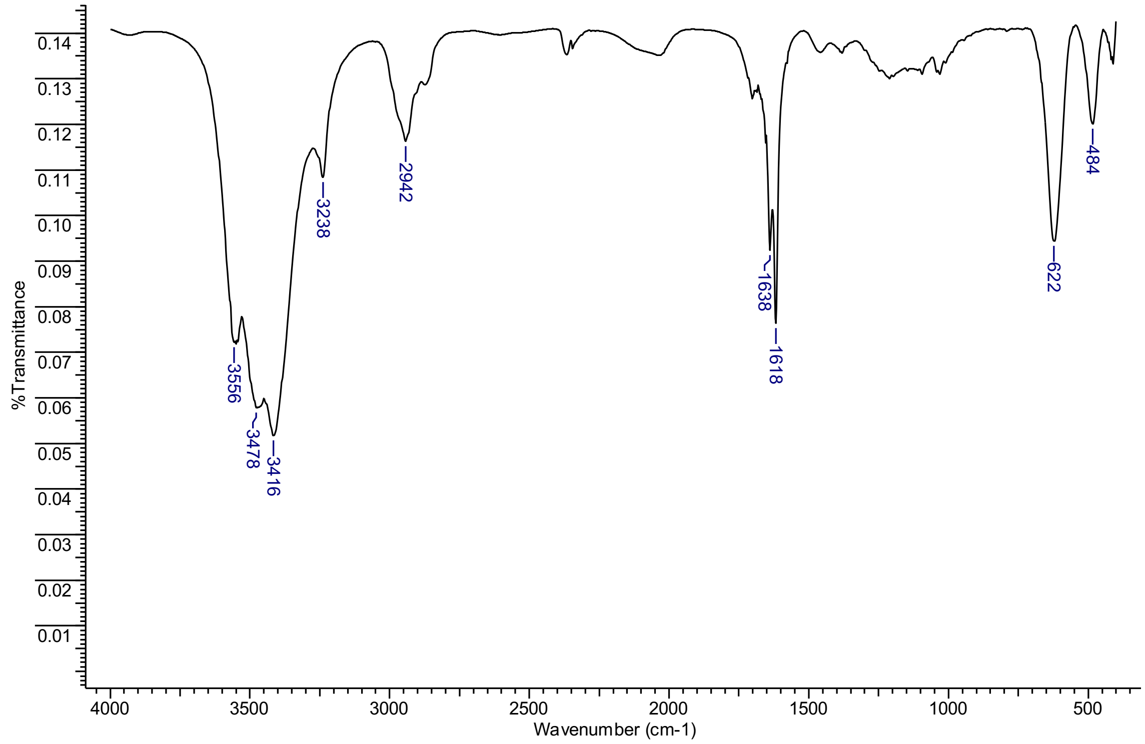

Figure S19. FTIR (KBr) spectrum of **P6**.

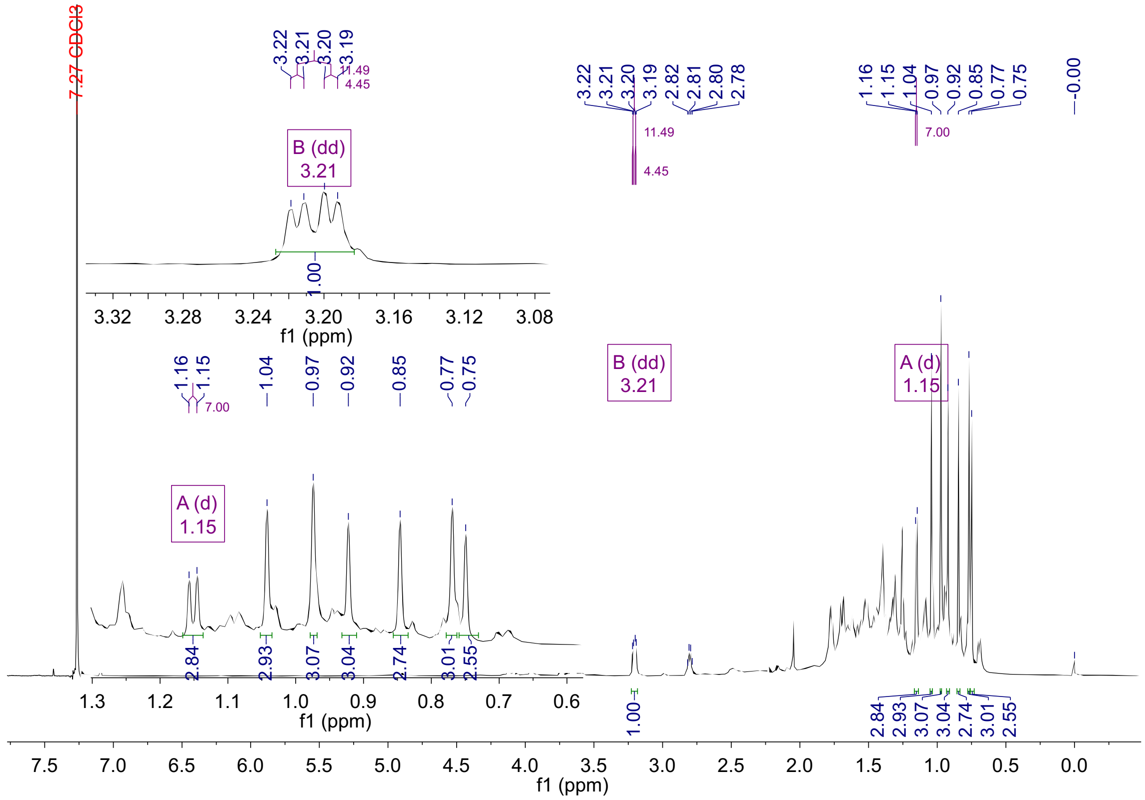

Figure S20. ^{1}H NMR spectrum (600 MHz, $CDCl_3$) of **P6**.

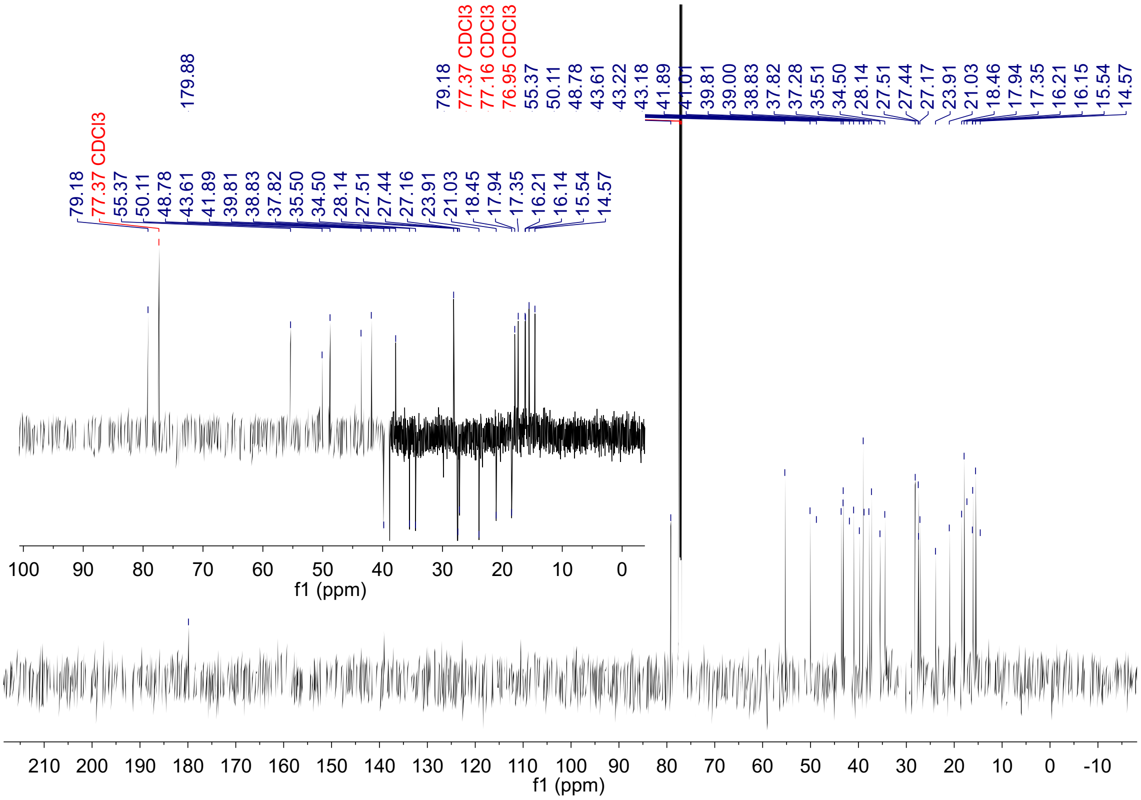

Figure S21. ^{13}C NMR and DEPT-135 spectra (150 MHz, $CDCl_3$) of **P6**.

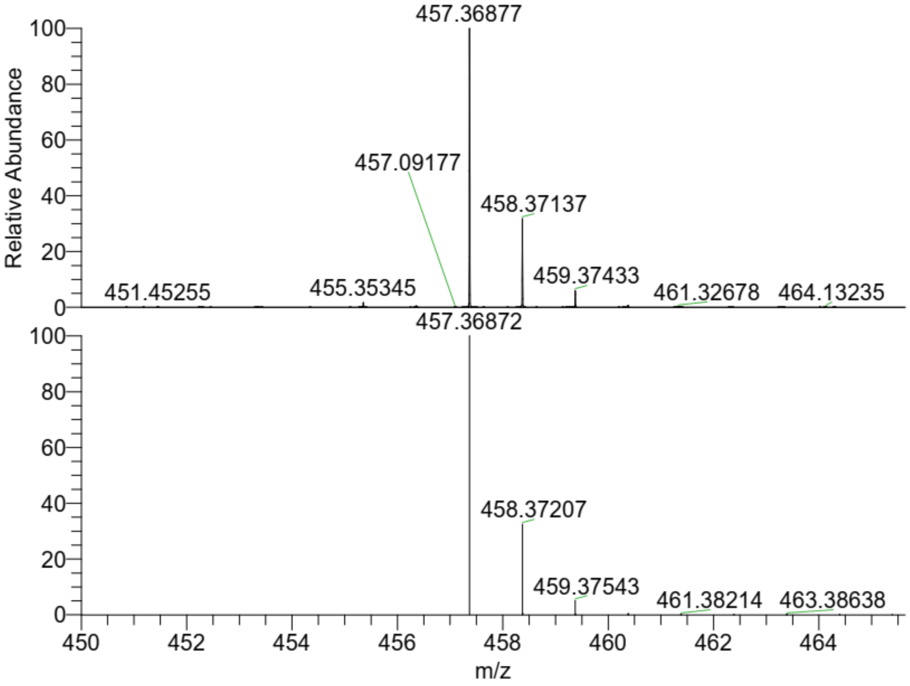

Figure S22. Mass spectrum (HR-H-ESI-MS) of **P6**.

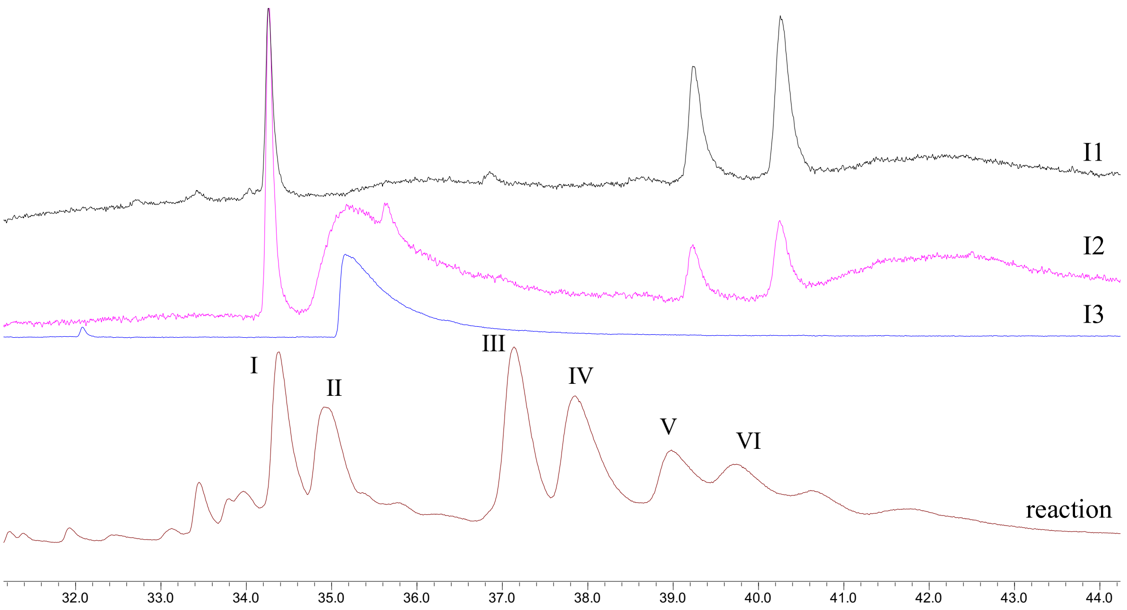

Figure S23. GC profile comparison of the reactions made with mannitol (**I1**), NaN3 (**I2**), BHT (**I3**), and the initial reaction.

Table S1. Retention indexes calculated for peaks I to VI of the reaction chromatogram – column RTX-5

**Compound/peak**	**Retention index**
Lupenone (I)	3405
Lupeol (II)	3441
29-norlupan-3,20-dione (III)	3592
3*β*-hydroxy-29-norlupan20-one (IV)	3641
P7 (V)	3718
P8 (VI)	3769
